# Comprehensive Analysis of Programmed Cell Death Signature in the Prognosis, Tumor Microenvironment and Drug Sensitivity in Lung Adenocarcinoma

**DOI:** 10.3389/fgene.2022.900159

**Published:** 2022-05-18

**Authors:** Shize Pan, Heng Meng, Tao Fan, Bo Hao, Congkuan Song, Donghang Li, Ning Li, Qing Geng

**Affiliations:** Department of Thoracic Surgery, Renmin Hospital of Wuhan University, Wuhan, China

**Keywords:** programmed cell death, lung adenocarcinoma, immunologic landscape, biomarker for immunotherapy response, immune subtype, ferroptosis

## Abstract

Programmed cell death (PCD) is a process that regulates the homeostasis of cells in the body, and it plays an important role in tumor immunity. However, the expression profile and clinical characteristics of PCD-related genes remain unclear. In this study, we comprehensively analysed the PCD genes with the tumor microenvironment (TME), drug sensitivity, immunothearapy response, and evaluated their prognostic value through systematic bioinformatics methods.We identified 125 PCD-related regulatory factors, which were expressed differently in lung adenocarcinoma (LUAD) and normal lung tissues. 32 PCD related prognostic genes associated with LUAD were identified by univariate Cox analysis. 23 PCD-related gene signature was constructed, and all LUAD patients in the Cancer Genome Atlas (TCGA) dataset were stratified as low-risk or high-risk groups according to the risk score. This signature had a powerful prognostic value, which was validated in three independent data sets and clinical subtypes. Additionally, it has unique properties in TME. Further analysis showed that different risk groups have different immune cell infiltration, immune inflammation profile, immune pathways, and immune subtypes. In addition, the low-risk group had a better immunotherapy response with higher levels of multiple immune checkpoints and lower Tumor immune dysfunction and exclusion (TIDE) score, while the high-risk group was sensitive to multiple chemotherapeutic drugs because of its lower IC50. In short, this is the first model to predict the prognosis and immunological status of LUAD patients based on PCD-related genes. It may be used as a predictor of immunotherapy response to achieve customized treatment of LUAD.

## Background

In recent decades, lung cancer has become a public health burden threatening global health, and its incidence has remained high (Zhang and Luo, 2021). In 2020, there are an estimated 2.2 million new cancer cases and 1.8 million deaths worldwide. Its incidence is second only to female breast cancer and is the main cause of male cancer morbidity and mortality (Sung and Ferlay, 2021). Among all primary types of lung cancer, non-small cell lung cancer accounts for about 85%, which includes adenocarcinoma, squamous cell carcinoma, bronchoalveolar adenocarcinoma, large cell carcinoma, and other pathological types (Torre and Bray, 2015). Among them, LUAD accounts for more than 40% of lung cancers and is also a major pathological subtype of lung cancer (Abe and Tanaka, 2016). For many years, lung cancer genesis mechanism and potential treatment methods have always been the endpoints of lung cancer research. A large amount of investment has been made in clinical treatment strategies for lung cancer, such as molecularly targeted drugs and immune checkpoint blockades (ICBs). However, despite the remarkable research, the 5-years survival rate for lung cancer is still only 17% (Zhao and Varn, 2018). Therefore, novel prognostic methods to identify high-risk patients are required to further assist in the design of new therapeutic options for LUAD patients. With the development of bioinformatics, there had been many PCD-related prognosis signatures to stratify lung adenocarcinoma in the past (Dong and Bian, 2021; Zhang and Yang, 2021). However, these signatures constructed the model using just a single cell death-related gene, ignoring the inner association or cross-talk in PCDs. As a consequence, including all PCD-related genes into the model may be more persuasive.

PCD is an essential process that is vital for the growth and development of an organism. There are five classical cell death pathways: cell death associated with autophagy, apoptosis, necrosis, pyroptosis, and ferroptosis (Dixon and Lemberg, 2012; Vande Walle and Lamkanfi, 2016; Chen and Kang, 2018; Green, 2019). It has been demonstrated that these PCDs are interconnected and can be cross-regulated with each other. Autophagy, also known as type II programmed cell death, plays a role in the onset and progression of a variety of illnesses (Su and Mei, 2013). Autophagy and apoptosis have a complicated relationship. Autophagy can block apoptosis in some instances, but it can also trigger cell death in combination with apoptosis or on its own as a backup mechanism in the case of apoptosis deficit (Su and Mei, 2013). Some drug studies targeting the autophagy pathway have shown that promoting autophagic death or inhibiting autophagy protection is an effective measure to eliminate tumor cells and resist drug resistance to chemotherapy in recent years, providing a glimmer of hope for overcoming the difficult problem of the tumor (Baraz and Cisterne, 2014). Both apoptosis and pyroptosis are involved in the activation of caspase family members, and they may have a common evolutionary origin. The latest results also suggest that gasdermin E (GSDM-E) is homologous to gasdermin D (GSDM-D) in structure and pore-forming function, and can be cleaved and activated by caspase-3, the executor of apoptosis (Jiang and Qi, 2020). The solubility and inflammatory morphology of necroptosis and pyroptosis are similar, resulting in a cross-over of both processes. NLRP3 inflammatory bodies can produce pyroptosis and can be triggered when cellular ion homeostasis changes; however, this trait also permits it to be activated in response to necroptosis-induced membrane destruction (Frank and Vince, 2019). Ferroptosis is a form of cell death caused by uncontrolled peroxidation of phospholipid membrane caused by iron dependence. NCOA4 can play a role in the degradation of ferritin (cellular iron storage protein) and support ferroptosis. Blocking autophagy or knocking out NCOA4 can inhibit the accumulation of labile iron and ROS associated with ferroptosis and prevent eventual ferroptosis (Zhou and Liu, 2020). These results indicate that these PCDs not only regulate each other in function but also have crosstalk with each other in the process of tumor development (Tang and Xu, 2020).

As early as the 1990s, the important relationship between PCD and the immune system was valued. In healthy people, the removal of thymocytes that express autoreactive or non-reactive T cell receptors (TCRs), the removal of autoreactive immature B cells, and the regression of inflammation are inseparable from PCD(Nagata and Tanaka, 2017). When PCD in the body is abnormal, the immune system will also change accordingly, such as apoptosis leading to immune deficiency (Rieux-Laucat and Le Deist, 1995; Madkaikar and Mhatre, 2011). As one of the important regulation methods of the body’s immune system, PCD may have potential biological significance in tumor treatment (Zhang and Zhang, 2020). However, the role of PCD in the immune microenvironment of LUAD has not been thoroughly studied.

In this study, we explored the prognostic significance of PCD genes and developed a new risk score based on the PCD related genes, which had a powerful value to predict the prognosis of LUAD. Investigating the immune cell infiltration, TME, and drug sensitivity in different risk groups, it is possible to predict immunotherapy response and find sensitive drugs for patients with LUAD in different risk groups.

## Materials and Methods

### Publicly Data Collection

The entire flow chart of this study was shown in Supplementary Figure S1. Cases with LUAD from four public databases were enrolled in this study. Among them, 368 LUAD samples with clinical characteristics were collected from TCGA(https://portal.gdc.cancer.gov/), which served as the training set. The other three independent validation sets containing 1067 cases were downloaded from Gene Expression Omnibus (GEO) (http://www.ncbi.nlm.nih.gov/geo), including226 samples from GSE31210, 398 samples from GSE72094, and 443 samples from GSE68465. Log2 conversion was performed for mRNA expression data, and the average expression amount was taken as the gene expression quantity. The basic clinical characteristics of these four cohorts are shown in Table 1.

**TABLE 1:** Clinical characteristics of the patients from multiple institutions.

Characteristics	TCGA N = 368	GSE31210 N = 226	GSE68465 N = 443	GSE72094 N = 398
Age	65.06 ± 0.5062	59.57 ± 0.4924	64.42 ± 0.4799	69.36 ± 0.4736
Gender				
Male	172 (46.7%)	105 (46.5%)	223 (50.3%)	176 (44.2%)
Female	196 (53.3%)	121 (53.5%)	220 (49.7%)	222 (55.8%)
Smoking				
Yes	252 (68.5%)	111 (49.1%)	300 (67.7%)	300 (75.4%)
No	116 (31.5%)	115 (50.9%)	49 (11.1%)	31 (7.9%)
NA	0	0	94 (21.2%)	67 (16.8%)
Stage				
I and II	309 (84.0%)	226 (100%)	NA	321 (80.7%)
III and IV	59 (16.0%)	0	NA	72 (19.3%)
Status				
Alive	304 (82.6%)	191 (84.5%)	236 (53.3%)	113 (28.4%)
Death	64 (17.4%)	35 (15.5%)	207 (46.7%)	285 (71.6%)

### The Identification of PCDs Mode Gene Sets

Apoptosis and necroptosis, these two gene sets related to programmed cell death mode were downloaded from Gene Set Enrichment Analysis (GSEA) (http://www.gsea-msigdb.org/gsea). The gene set of autophagy was downloaded from Human Autophagy Database (HADb) (http://www.autophagy.lu/index.html) The gene set of pyroptosis was referenced in the following literature reports (Wang and Yin, 2017; Karki and Kanneganti, 2019; Xia and Wang, 2019). The gene set of ferroptosis was downloaded from FerrDb (http://www.zhounan.org/ferrdb). We identified the DEGs between normal and malignant tissues in TCGA samples. The DEGs were identified using the “limma” package with *p*-value <0.05. Using the search tool of Retrieval of Interacting Genes (STRING), the network of DEG was constructed.

### The PCD Signature Generation in LUAD

The DEGs were selected from the TCGA validation set for univariate cox regression analysis. *p* <0.05 was selected as the *p*-value, and 32 prognosis-related genes were obtained by using univariate Cox analysis. Then lasso regression was used to narrow the candidate genes and construct a prognostic model. Finally, the 23 genes and their regression coefficients were retained by multivariable Cox regression analysis. Then, the risk score was generated according to the following formula. Risk score = β1× G1+ β 2 × G2 + …β n × Gn, where βn represented the coefficient of the gene, and Gn presented the expression level of the gene. The samples in the training and validation sets were divided into the high and low-risk groups based on the optimal cut-off value of the risk scores. Detailed methods of this part could be found in the previous article (Fan and Pan, 2021).

### Pathway and Function Enrichment Analysis

Kyoto Encyclopedia of Genes and Genomes (KEGG) and Gene Ontology (GO) pathway and functional enrichment analysis were performed using R statistical software and R packages.

### Analysis of Immune Cell Infiltration, Immune Pathway, and TME

To describe the immune landscape of LUAD, CIBERSORT(Newman and Liu, 2015) and single sample GSEA (ssGSEA) (Barbie and Tamayo 2009) analysis were used to quantify the infiltration abundance of various immune cells and immune pathways in TME. The ESTIMATE algorithm (Becht and Giraldo, 2016) was used to estimate the content of Stromal and Immune cells in malignant tumors, and to infer tumor purity and calculate immune score and stromal score.

### GSVA and GSEA Analysis

The results of the seven metagenes clusters were emulated by Gene Sets Variation Analysis (GSVA), which evaluates whether a gene is highly or lowly expressed in a sample in the context of the sample population distribution (Hänzelmann et al., 2013). Signaling pathways related to the PCD-based signature were analyzed through Gene Set Enrichment Analysis (GSEA). GSEA is commonly used to evaluate the distribution trend of genes in a predefined gene set, which has been widely reported to investigate the biological process difference between subtypes (Hu and Wu., 2021; Zhang and Qin, 2021).

### Tumor Mutational Burden and Neoantigen Analysis

Gene mutation data of patients with LUAD was generated from the TCGA dataset (https://portal.gdc.cancer.gov/). The definition of tumor mutational burden (TMB) is mutations per million bases. The protein with specific amino acid sequence variation produced by cancer cells based on genetic variation is called“neoantigen”. We obtained neoantigen data of LUAD patients from The Cancer Immunome Atlas (TCIA) (https://tcia.at/home).

### TIDE and Immune Checkpoint Analysis

TIDE score was first defined by Jiang and his colleagues (Jiang and Gu, 2018), which has been proven to have robust power for predicting the prognosis and immunotherapy response of cancer patients. We obtained the TIDE score, IFN-g (IFNG), merck18 (T-cell-inflamed signature) score, CD8 score, dysfunction score, and exclusion score from the TIDE web (http://tide.dfci.harvard.edu). The expression of immune checkpoints (PD-1, PD-L1, CTLA4, TIM-3, and LAG3) was extracted from the TCGA database.

### Statistical Analysis

The patients with LUAD were divided into high- and low-risk groups according to the optimal cutoff value. The Kaplan–Meier method was used to evaluate the OS between the high- and the low-risk group, and the log-rank was used to verify the significant difference. The unpaired u-test was used to analyze the distribution of immune cells, TMB, number of neoantigens, number of clonal neoantigens, number of sub-clonal neoantigens, PD-L1 protein expression, and TIDE in the different risk groups. Independent prognostic factors were calculated by Cox proportional hazard regression model. Among all the analysis methods, *p* < 0.05 was considered statistically different. R 3.6.1 (https://www.r-project.org) and GraphPad Prism 8.0.1. were used to analyze data and create tables and figures.

## Results

### Identification of Programmed Cell Death Mode in LUAD

PCD pathways include five specific cell death modes, namely apoptosis, necroptosis, autophagy, pyroptosis, and ferroptosis. We analyzed the cell death mode gene sets from different sources, and the sizes of the five PCD pathway gene sets are not consistent, 568 genes for apoptosis, 52 genes for necroptosis, 328 genes for autophagy, 33 genes for pyroptosis, and 258 genes for ferroptosis. The Venn diagram shows that the five PCD pathway genes partially overlap, suggesting that there is crosstalk in the PCD pathway (Supplementary Figure S2A). Heatmap (Supplementary Figure S2B) showed the expression characteristics of each gene of PCD. Then we selected two-fold at least DEGs to draw a gene regulatory network in all PCD genes (Supplementary Figure S2C), which indicates there is cross-talk in these PCD genes.

### Features of Consensus Matrix Post-clustering in LUAD

**FIGURE 1:**
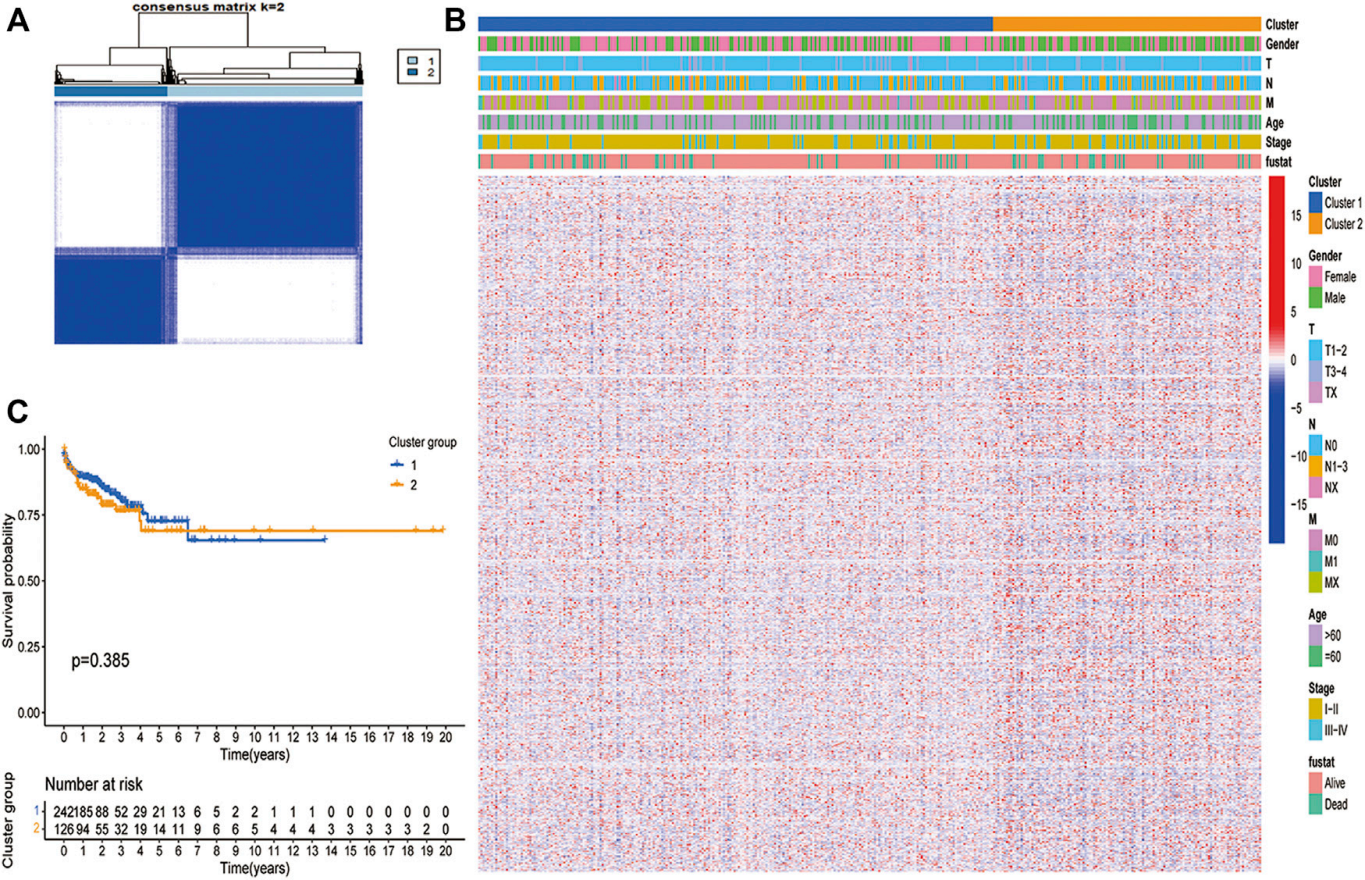
Unsupervised clustering analysis in TCGA dataset. **(A)** The TCGA dataset were stratified into two clusters based on the consensus matrix (k = 2). **(B)** Differences in clinicopathologic features and expression levels of differentially expressed PCDs between the two distinct clusters. (blue: low expression level; red: high expression level). **(C)** Survival curve analysis show no difference in Os between the two clusters (*p* > 0.05).

Subsequently, we use unsupervised clustering analysis to cluster the LUAD data into cluster 1 and cluster 2 (Figure 1A). Heatmap (Figure 1B) showed the difference in gene expression between the two clusters. It can be seen that the most of gene expression of cluster 2 is higher than that of cluster 1. However, these two clusters did not show a clear difference in survival (Figure 1C).

### The Landscape and Prognostic Significance of the PCD-Based Signature in LUAD

**FIGURE 2:**
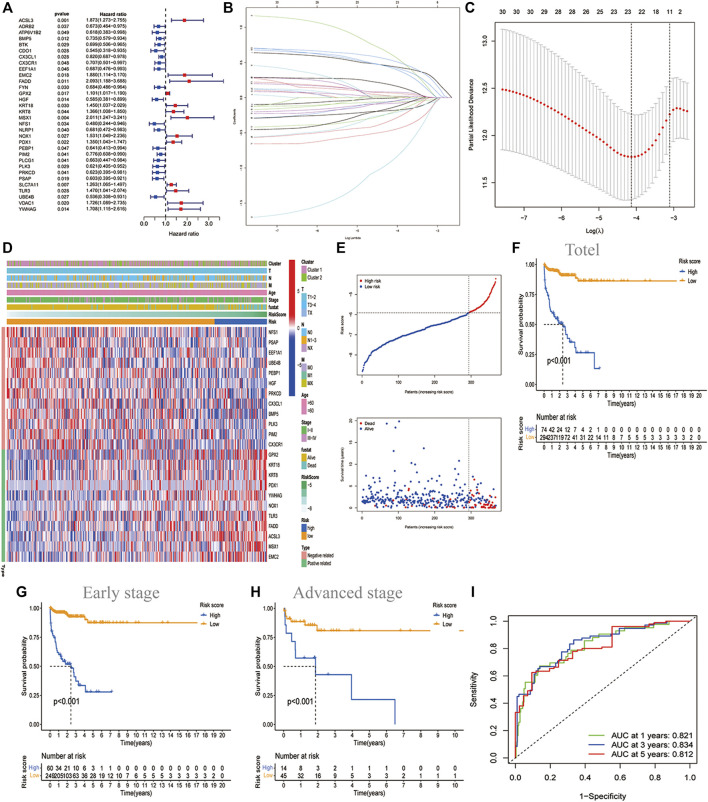
Construction of the PCD-based signature in TCGA datasets. **(A)**The forest map showing the 32 prognostic PCD-related DEGs by univariate cox regression analysis (*p* < 0.05). **(B)** Cross-validation for adjusting the parameter selection in the LASSO regression. **(C)** LASSO regression analysis of the 32 OS-related DEGs. **(D)**Heatmap showing the features of 23 identified genes and clinical characteristics of two molecular clusters. **(E)** The distribution of risk score and survival states. Kaplan Meier plot showing the significant difference of OS in total LUAD (*n* = 368) **(F)** ; in early-stage (I or II) (n = 309) **(G)**and in advanced stage (III or IV) (n = 59) **(H)** in the high-risk and low-risk groups. **(I)** ROC curve showing the sensitivity and specificity to predict 1-, 3-, and 5-years survival based on PCD-based signature, with the area under curve being 0.8210.834, and 0.812, respectively.

32 prognostic related genes were screened out by univariate regression analysis for further analysis (Figure 2A). Lasso regression analysis was performed on these genes to narrow the candidate genes (Figures 2B,C). Finally, we selected 23 genes to construct a PCD-related signature with the risk score = 0.4460 ACSL3 +(-0.05857 BMP5)+(-0.0820 CX3CL1)+(-0.0062 CX3CR1)+(-0.3164 EEF1A1)+(0.6089 EMC2) +(0.4358 FADD)+(0.0208 GPX2)+(-0.1823 HGF)+(0.0215 KRT18)+(0.0217 KRT8)+(0.5293 MSX1)+(-0.8964 NFS1 )+(0.1788 NOX1 )+(0.0659 PDX1)+(-0.2112 PEBP1 )+(-0.0517 PIM2)+(-0.0541 PLK3) +(-0.1423 PRKCD )+(-0.46223 PSAP )+(0.4212 TLR3 )+(-0.2217 UBE4B)+(0.0794 YWHAG). Figure 2D showed that the distribution of these 23 genes was significantly different in different risk groups. Additionally, we depicted the copy number variation (CNV) profiles in the human chromosome and frequency of somatic mutations of these 23 genes and found that HGF presented the highest mutation frequency, followed by UBE4B (Supplementary Figure S3). The samples with LUAD were divided into two risk groups based on the cut-off value of the risk score. Among them, the high-risk group had a higher proportion of death samples, while the low-risk group had more survivors (Figure 2E). Principal component analysis (PCA) and t-distributed stochastic neighbor embedding analysis (t-SNE) showed that LUAD can be well divided into two categories according to the risk score, which indicated that the PCD genes-based signature had good stratification ability (Figure 2E). This was fully verified by the later survival analysis, with the overall survival (OS) of the high-risk group being significantly lower than that of the low-risk group (*p* < 0.001, Figure 2F), regardless of whether the patients were in early-stage (I or II) or advanced stage (III or IV) (Figures 2G,H). By performing a time-dependent receiver operating characteristic (ROC) analysis to evaluate the model’s sensitivity and specificity (Figure 2I), we noticed that the area under the ROC curve was 0.821, 0.834, and 0.812 for 1 year, 2 years, and 3 years, respectively, suggesting that PCD-related gene sets were valuable for the diagnosis of LUAD. Meanwhile, we also used three independent GEO data sets for confirmatory ROC analysis indicating that our PCD-based signature had a good prognostic ability (Supplementary Figure S4).

### Validation of the Risk Model in the GEO Cohort

**FIGURE 3:**
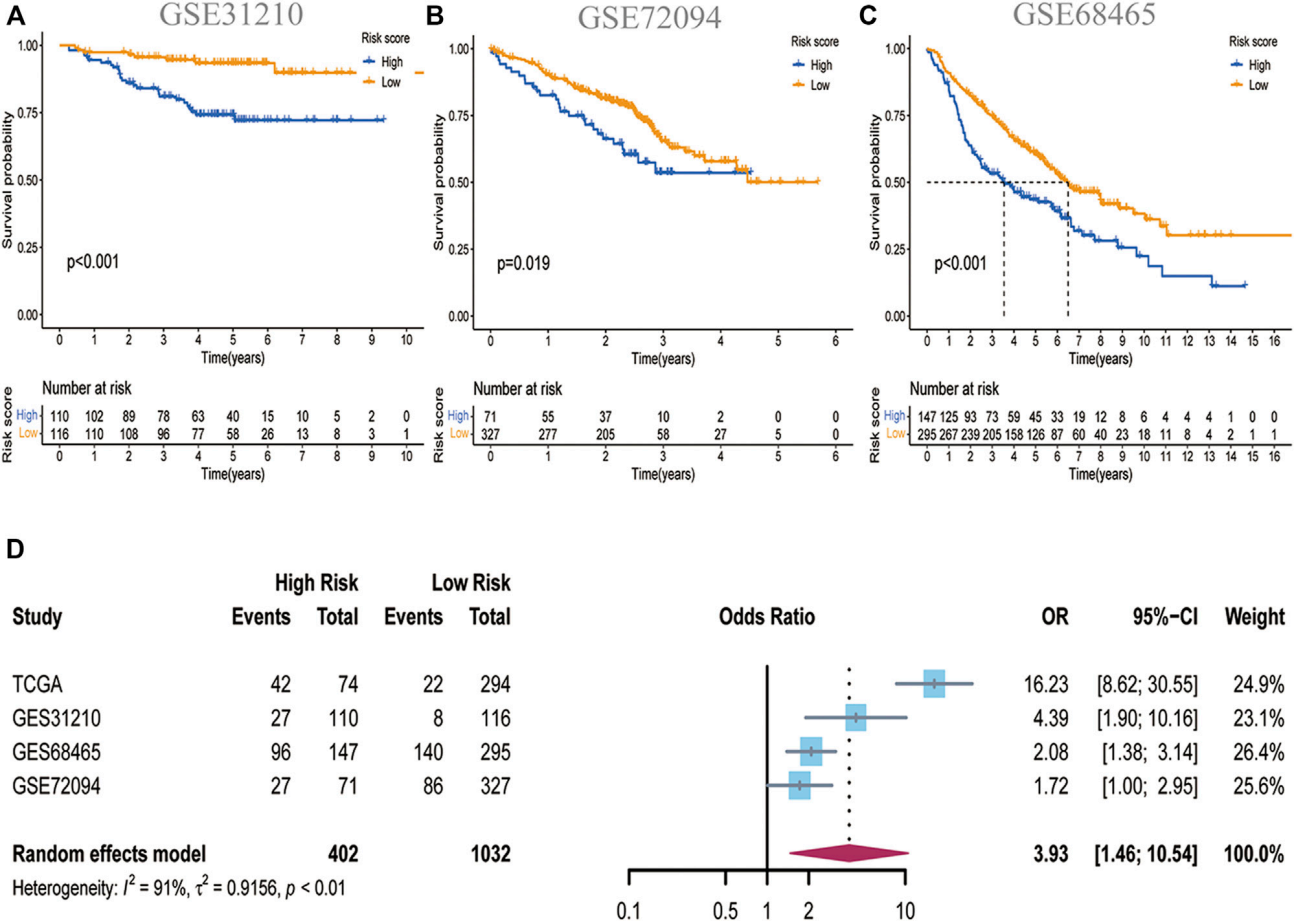
Validation of the PCD-related signature in GEO datasets. **(A)**Kaplan-Meier curves showing the significance of OS in GSE31210 (n = 226) **(B)** in GSE 72094 (n = 398) and **(C)** in GSE68465 (n = 443). **(D)**A meta-analysis showed the prognostic outcomes in four independent datasets (HR:3.93; 95%CI:1.46–10.54).

We enrolled three GEO sets for external validation to validate our risk model’s forecasting capacity. Patients in the three GEO validation sets were separated into two groups based on the optional cut-off value of their risk score. Comparing the OS time of the high-risk and low-risk groups in GES31210, it was found that the OS of the low-risk group was significantly higher than that of the high-risk group (Figure 3A). GSE72094 and GSE68465 yielded the same results (Figures 3B,C). In addition, we determined the prognostic significance of PCD-related signature in these public cohorts through a prognostic meta-analysis based on these four groups (n = 1,434). Our results confirmed that PCD-related signature was a risk factor for LUAD patients (OR, 3.93; 95%CI, 1.46–10.54, *p* < 0.01) (Figure 3D). To demonstrate the prognostic ability of this RCD-related signature, we investigated the effect of risk groups on OS in various clinical subtypes and noticed that the low-risk group had better OS than the high-risk group no matter whether the patients were old or young, male or female, in early-stage T stage (T1-2) or advanced T stage (T3-4), in N0 or N1-3 stage. For the M1 stage, there is no obvious OS difference between the two groups, and probably due to too few cases (Supplementary Figure.S5).

### The PCD-Related Gene Signature Is an Independent Risk Factor for LUAD

Univariate and multivariate regression analyses were undertaken to confirm whether the PCD-related gene signature was influenced by other clinical parameters, and the findings revealed that risk score was an independent prognostic predictor (*p* <0.001, HR: 4.0072, 95%CI: 2.7365–5.8682. Table 2)

**TABLE 2:** Univariable and multivariable Cox regression analysis of the PCDs-based signature and clinical feature in TCGA dataset.

Variable	Univariable Analysis			Multivariable Analysis		
	HR	95%CI	*p* Value	HR	95%CI	*p* Value
Stage						
I-II or III-IV	1.8743	0.9612–3.6548	0.0652			
T Stage						
T1-2 or T3-4	2.7935	1.3750–5.5754	0.0045	1.2850	0.6181–2.6712	0.5019
N stage						
N0 or N1-3	1.8613	1.0145–3.4148	0.0448	2.0176	1.0897–3.7356	0.0255
M stage						
M0,M1 or Mx	2.1131	0.6484–6.8869	0.2146			
Gender						
Male or Female	1.6878	0.9100–3.1376	0.0966			
Age						
≤60 or ＞60	1.5049	0.8209–2.7588	0.1862			
Smoking						
Yes or No	0.6571	0.3563–1.2119	0.1788			
Risk Score						
High or Low	3.9516	2.7510–5.6760	<0.001	4.0072	2.7365–5.8682	<0.001

Abbreviations: HR, hazard ratio; CI, confidence interval.

### Biological Pathways of the PCD-Related Gene Signature

**FIGURE 4:**
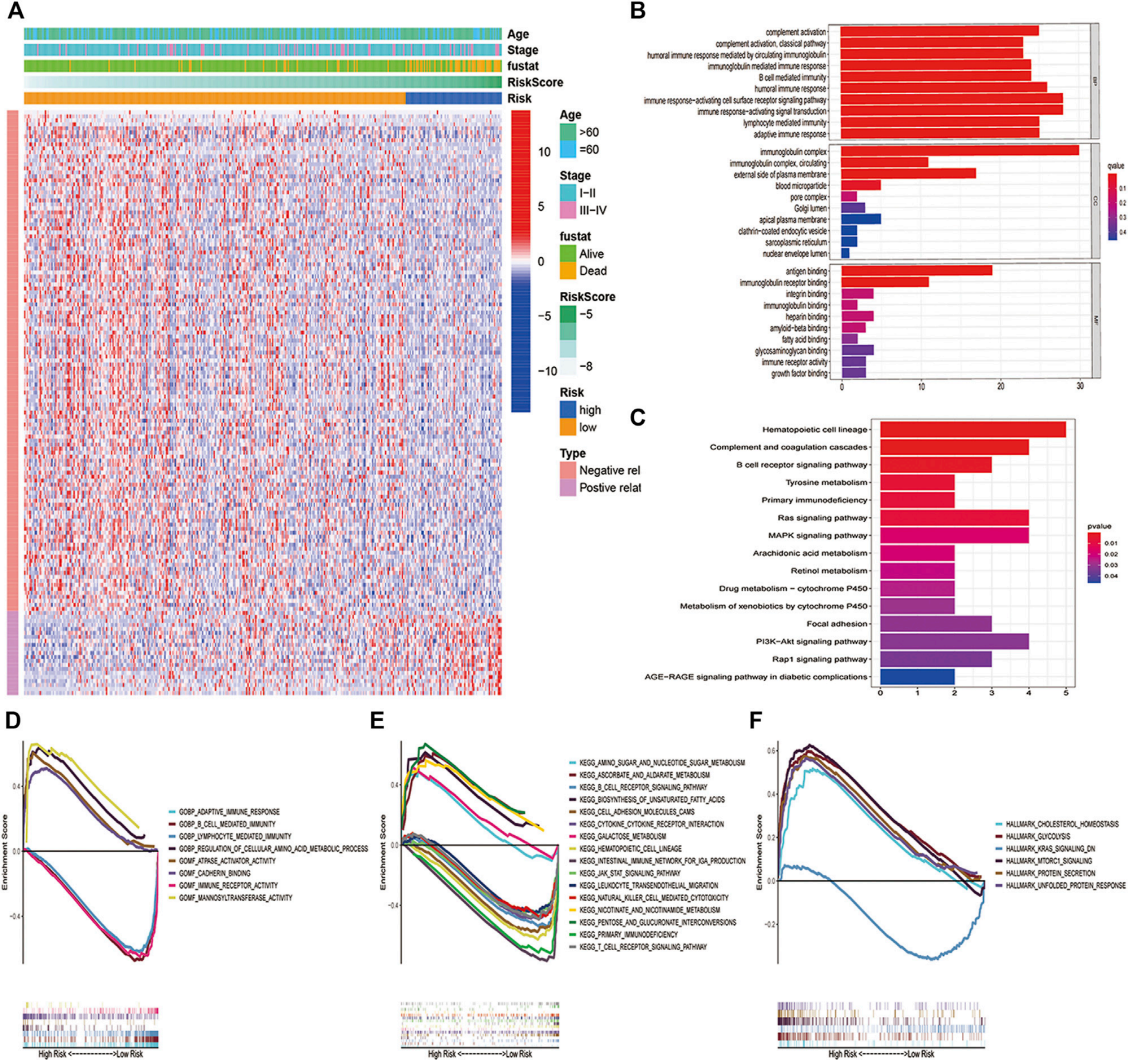
Biological pathway and function between the two risk groups. **(A)** Heatmap and the difference of clinical characteristics and DEGs between the high- and low-risk groups (blue: low expression level; red: high expression level). **(B)**GO and **(C)**KEGG analysis revealed that DEGs in high- and low-risk subgroups were enriched in multiple immune and tumor-related pathways. **(D–F)** GSEA analysis validated multiple biological pathways related to inmmunity and tumor enriched in the high-risk group and low-risk group.

For such a powerful predictive status, We paid our interest on the exploration of its potential mechanisms. Firstly, to analyze the molecular biological characteristics of this model comprehensively, we screened out these genes strongly related to PCD-related gene signature score [log FC(fold change)＞1, *p* < 0.05]. The heatmap (Figure 4A) indicated that 146 differentially expressed genes (DEGs) were selected, and most of them were positively related to high-risk groups and negatively related to low-risk groups. Then, for these genes, we further performed GO and KEGG function enrichment analysis to deeply study the mechanism. As shown in Figure 4B, these genes were mainly involved in many immune-related pathways, such as the B cell-mediated immunity pathway, immunoglobulin complex pathway, antigen binding, etc. In addition, KEGG analysis showed that these genes were closely related to hematopoietic cell lineage, B cell receptor signaling pathway, primary immunodeficiency, Ras pathway, MAPK pathway, etc. (Figure 4C). GSEA analysis (Jiang and Gu, 2018) was performed to prevent removing any genes that have vital biological roles but are not differentially expressed in the high-risk and low-risk groups. It was discovered that a variety of immunological pathways were correlated with a risk score, with strong enrichment in the low-risk group (Figures 4D–F).

### Inflammatory and Immunologic Profile of the PCD-Related Gene Signature

**FIGURE 5:**
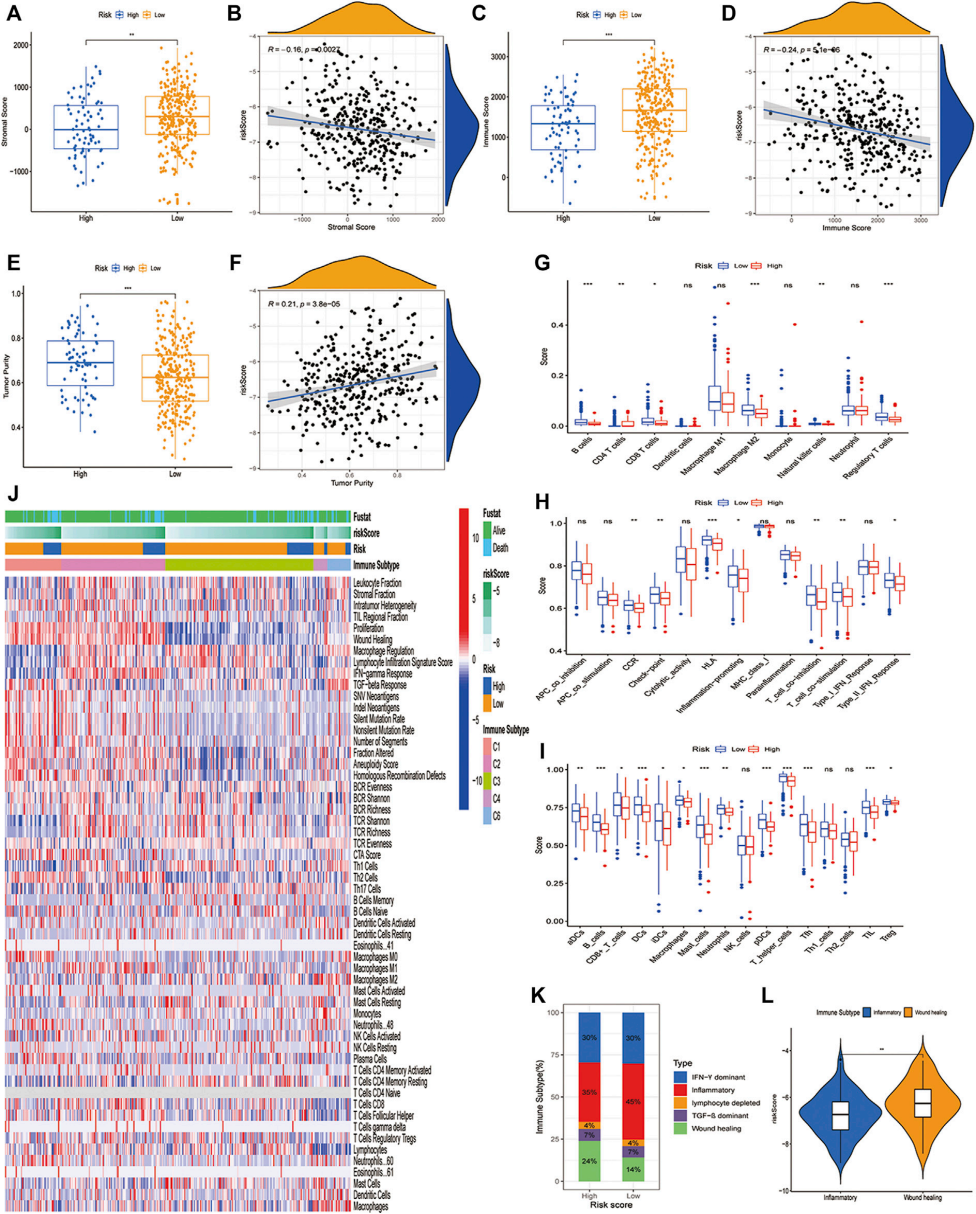
The immune landscape of PCD-related signature in LUAD. **(A,B)** Significant differences of stromal score in high- and low-risk groups and correlation with risk score. **(C,D)** Significant differences of immune score in high- and low-risk groups and correlation with risk score. **(E,F)** Significant differences of tumor purity in high- and low-risk groups and correlation with risk score. **(G)** Comparison of 10 types of immune cells in high- and low-risk groups through CIBERSORT. **(H,I)** Comparison of 16 types of immune cells and 13 immune-related pathways in high-risk and low-risk groups through ssGSEA. **(J)**The heatmap showing different cell type panels in different immune subtypes of lung cancer. **(K)** Estimated immune subtype proportion in high-risk and low-risk groups. **(L)** Comparison of the risk score in inflammatory subtype and wound healing subtype. (*, **, ***, and **** represent *p* < 0.05, *p* < 0.01, *p* < 0.001 and *p* < 0.0001, respectively).

Given that the identified characteristics are closely related to a series of immune pathways, we further studied the immune landscape of the LUAD high-risk and low-risk groups in the TCGA dataset. Firstly, we conducted the stromal score, immune score, and tumor purity in high-risk and low-risk groups. As we can see, compared to the low-risk group, the high-risk group had a lower stromal score (Figure 5A) and immune score (Figure 5C), but a higher tumor purity (Figure 5E). We conducted a correlation analysis between stromal score, immune score, tumor purity, and risk score. Only the tumor purity (Figure 5F) was positively correlated with the risk score, and the stromal score (Figure 5B) and immune score (Figure 5D) were negatively correlated with the risk score. This meant that the high-risk group was related to higher tumor purity and poorer immune infiltration environment. Secondly, we analyzed how immune cells and immunological pathways were enriched in the high- and low-risk groups. LM22 in CIBERSORT was used to evaluate immune cell infiltration in the high-risk and low-risk groups. The low-risk group had higher levels of B cells, CD8^+^ T cells, M2-type macrophages, natural killer cells, and regulatory T cells. Only CD4^+^ cells have a better infiltration proportion in the high-risk group (Figure 5G). To make our results more convincing, we also used the ssGSEA method to calculate the enrichment of 16 immune cells and 13 immune pathways in different risk groups. The results showed that the low-risk group had higher levers in B cells, DC cells, mast cells, pDC cells, T helper cells, Tfh cells, and TILs (Figure 5H). Besides that, we examined the relationship between CD8^+^ T cells, cancer-associated fibroblasts (CAFs), and myeloid-derived suppressor cells (MDSCs) and risk score, and discovered that MDSCs cells was highly expressed in the high-risk group and had a significant negative correlation with a risk score, while CD8^+^ T cells had an opposite trend (Supplementary Figure.S6). Interestingly, seven immunological pathways in the high-risk group were less active than those in the low-risk group (Supplementary Figure S5I). Finally, we further investigated the relationship between our risk signature and the immune subtypes, including wound healing, IFN-γ dominant, inflammatory, lymphocyte, immunologically quiet, and TGF-β dominant (Thorsson and Gibbs, 2018). The heatmap (Figure 5J) showed the landscape of immune cells and pathways in different immune subtypes. It was discovered that the low-risk group had more inflammatory subtypes, whereas the high-risk group had more wound healing subtypes (Figure 5K). Intriguingly, the Would healing group had a higher risk score than the Inflammatory group (Figure 5L).

### Relationship Between the PCD-Related Gene Signature and Inflammatory Metagenes in LUAD

**FIGURE 6:**
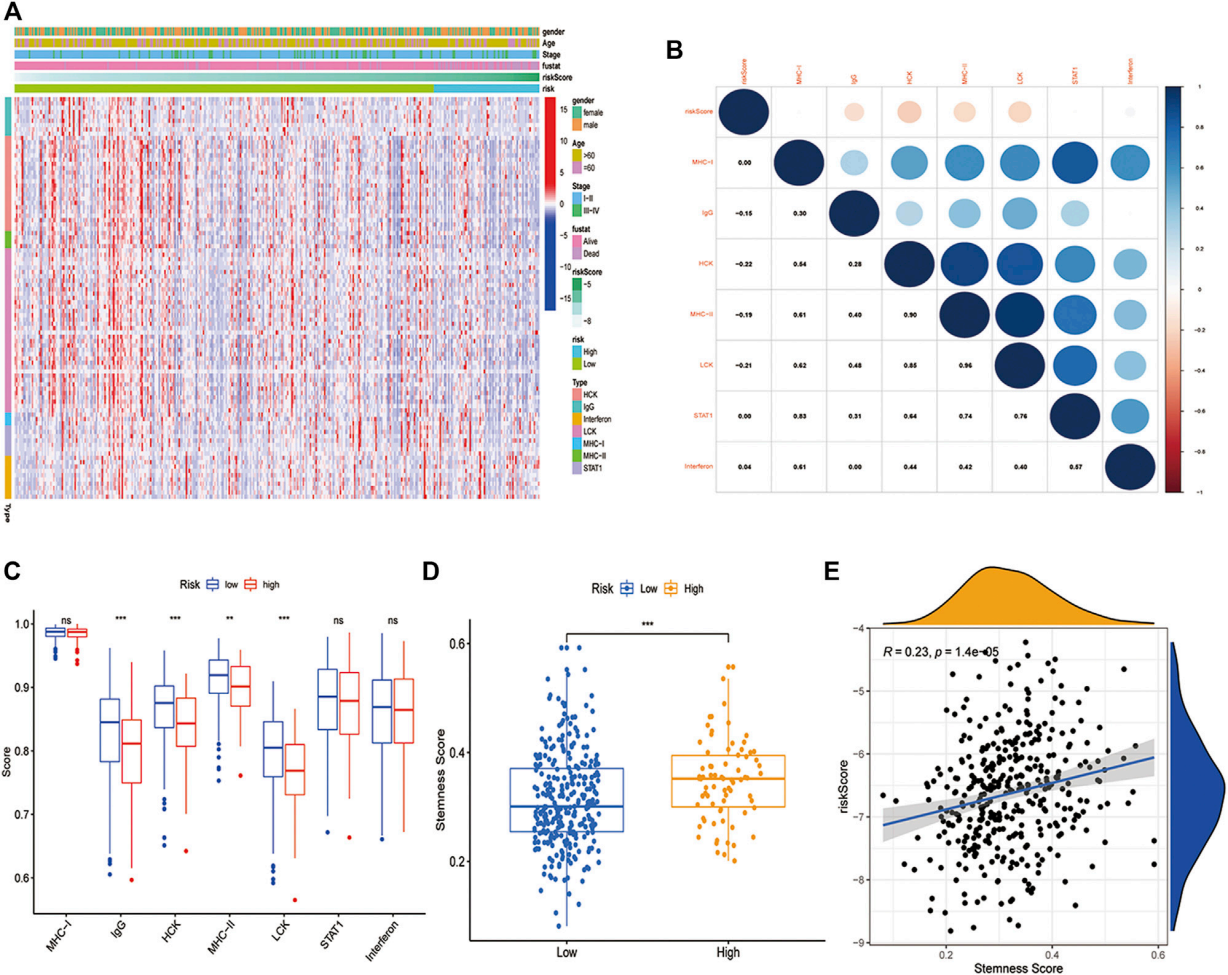
Inflammatory and immunologic profile of the PCD-related signature in TCGA cohort. **(A)** Heatmap showed the gene expression of immune-inflammatory metagenes in the two risk groups (blue: low expression level; red: high expression level). **(B)** A correlogram was generated based on Pearson *p*-value between risk score and metagenes. **(C)** Comparison of the immune-inflammatory metagenes in high-risk and low-risk groups. **(D)** Comparison of the stemness score in high-risk and low-risk groups. **(E)** Correlation of stemness score and risk score (R = 0.23, *p* < 0.05). (*, **, ***, and **** represent *p* < 0.05, *p* < 0.01, *p* < 0.001 and *p* < 0.0001, respectively).

To further understand the immune-inflammatory microenvironment in different risk groups, we depicted the profile of seven metagenes in the high- and low-risk group (Figure 6A). All the seven metagenes were IgG, STAT, MHC-I, Interferon, MHC-II, HCK, and LCK(Rody and Holtrich, 2009). Subsequently, we evaluated the relationship between risk score and the metagenes and found that the risk score was negatively related to IgG, HCK, MHC-II, and LCK (Figure 6B). Additionally, the low-risk group showed higher enrichment in IgG, HCK, MHC-II, and LCK than the high-risk group (Figure 6C). Moreover, we also investigated the correlation between risk score and stemness score, which was downloaded from UCSC Xena. A higher stemness score indicated a poorer anticancer immunity (Miranda and Hamilton, 2019). And in our study, the high-risk group had a higher stemness score as we expected (Figure 6D), while the risk score was positively related to stemness score (Figure 6E).

### Relationship Between the PCD-Related Risk Model and Biomarkers of Immunotherapy

**FIGURE 7:**
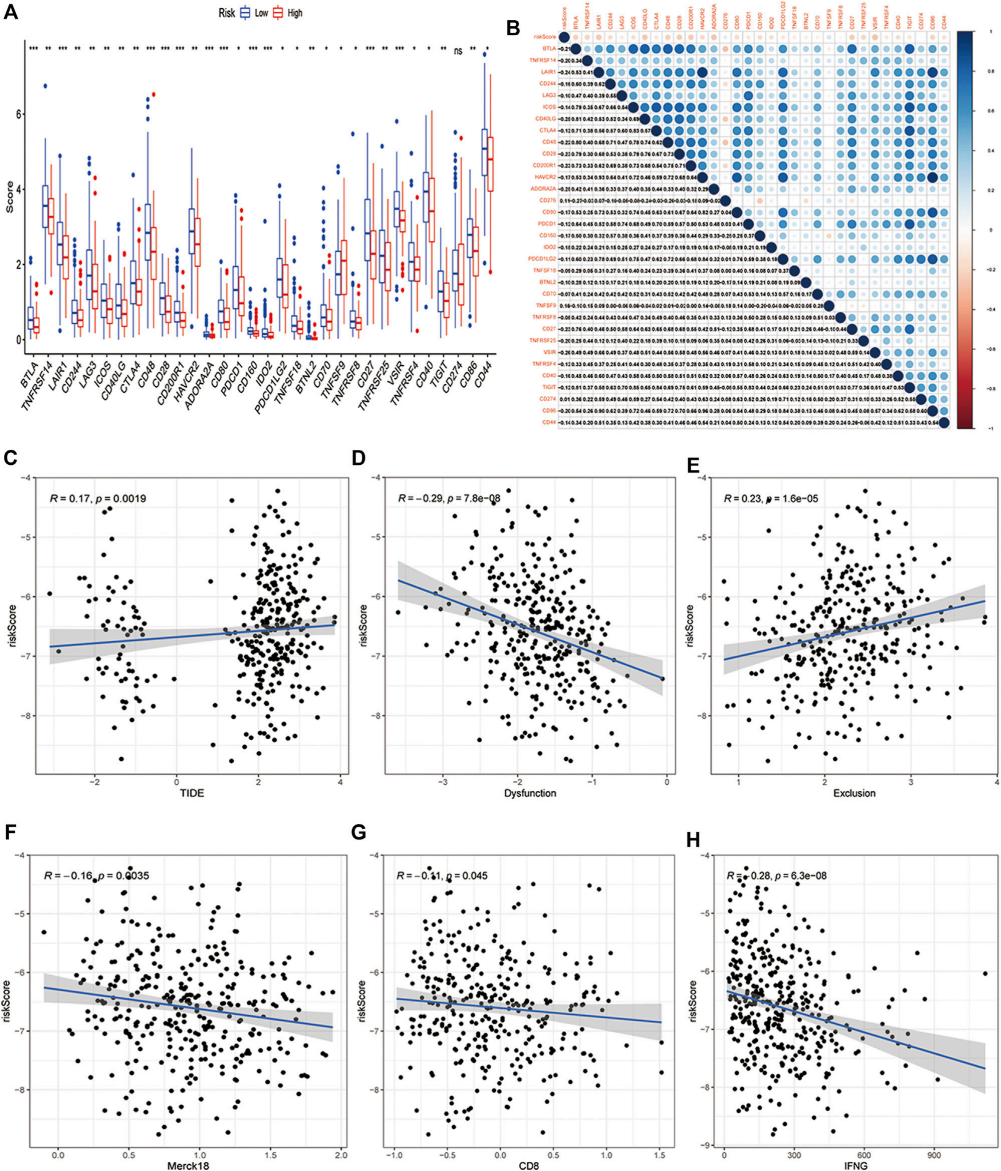
Relationship between the PCD-related signature and biomarkers of immunotherapy. **(A)** Comparison of the immune checkpoints in high-risk and low-risk groups. **(B)** A correlogram was generated based on the Pearson *p*-value between risk score and immune checkpoints. **(C)** Correlation of risk score and TIDE score, **(D)** T cells dysfunction score, **(E)**T cells exclusion score, **(F)** Merck18 score, **(G)** CD8, **(H)** IFNG in high-risk and low-risk groups. (*, **, ***, and **** represent *p* < 0.05, *p* < 0.01, *p* < 0.001 and *p* < 0.0001, respectively).

Immunotherapy that targets immune checkpoints is currently the first-line treatment for lung cancer, particularly in advanced tumors. PD1, PD-L1, TMB, LAG3, CTLA4, and TIM3 are being commonly used as immunotherapy response biomarkers (Havel and Chowell, 2019). Considering the important role of PCD signature in tumor immune inflammation and tumor immune microenvironment, we deeply explored the association between this PCD signature and immunotherapy response. Firstly, we compared immune checkpoint gene enrichment in different subgroups, and found that multiple genes were enriched in the low-risk group and negatively related to risk score (BTLA, TNFRSF14,LAIR1,CD244,LAG3,ICOS,CD40LG,CTLA4,CD48,CD28,CD200R1,HAVCR2,ADORA2A,CD80,PDCD1,CD160,IDO2,PDCD1LG2,TNFSF18,BTNL2,CD70,TNFRSF8,CD27,TNFRSF25,VSIR,TNFRSF4,CD40,TIGIT,CD274,CD86,CD44), only CD276 and TNFSF9 were enriched in the low-risk group and positively related to risk score, while PD-L1 (CD274) was not differentially expressed in the high- and low-risk groups (Figures 7A,B). TIDE is a more accurate biomarker than TMB and immune checkpoints because it replicates the two primary mechanisms of tumor immune escape: development of T cell dysfunction in tumors with high cytotoxic T lymphocyte (CTL) invasion and suppression of T cell invasion in cancers with low CTL levels (Jiang et al., 2018). T cell dysfunction score and T cell exclusion score, which were calculated based on the expression level of genes in a given gene set, were shown to have good prediction performance for ICB response (Jiang et al., 2018). The TIDE score, T-cell-inflamed signature (merck18), T-cell dysfunction score, T-cell exclusion score, IFNG score, and CD8 were obtained from the TIDE system. Subsequently, we deeply explored the relationship between TIDE and our risk signature, and the results showed that the PCD-based risk score was positively related to TIDE score and T cells exclusion score (Figures 7C,E), while negatively related to T cells dysfunction score, Merck 18, IFNG and CD8 (Figures 7D–H). The study has shown that patients with high TMB have better treatment outcomes with ICBs(Sholl and Hirsch, 2020). However, there was no correlation between neoantigen and risk score, and TMB did not differ between high- and low-risk groups (Supplementary Figure.S7-S8). Semi-inhibitory concentration (IC50) is an important index to evaluate the efficacy or response of drugs. Based on the sample transcriptome, we evaluated the IC50 of each sample using the “prrophetic” R package (Geeleher and Cox, 2014). As we expected, low-risk groups had a higher IC50 than high-risk groups in multiple chemical drugs, including Lapatinib, Imatinib, and so on, suggesting that the high-risk groups were sensitive to these drugs (Supplementary Figure.S9).

### Comparison of the Predictive Power of PCD-Related Signature With Other Biomarkers and Construction of a Nomogram in LUAD Datasets

**FIGURE 8:**
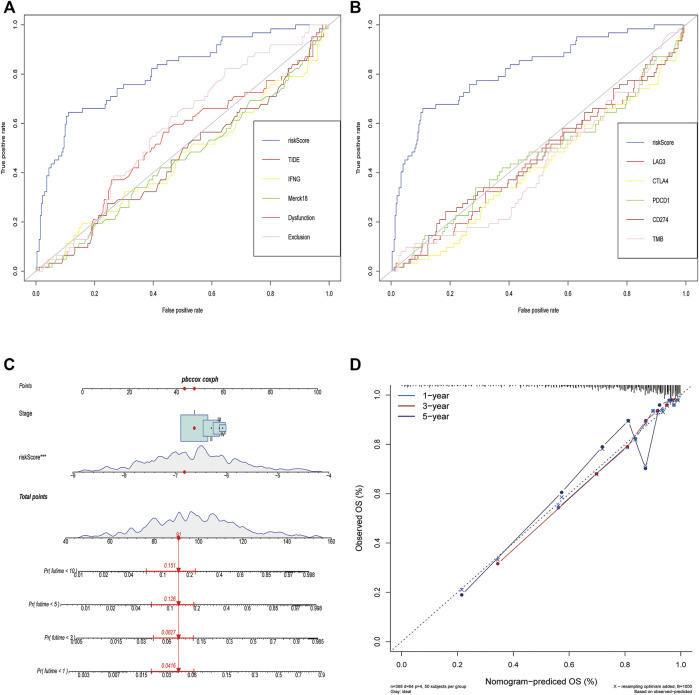
Comparison of the predictive power of PCD-related signature with other biomarkers and construction of a nomogram in TCGA cohort. **(A,B)** ROC curve compared the sensitivity and specificity of risk score and other biomarkers for predicting OS. **(C)** Nomogram combined the TNM staging and risk score for predicting the 1-, 3-, 5-, and 10-years OS. **(D)** Calibration curves of the nomogram for predicting of 1-, 3-, and 5-years OS.

The PCD-based signature was strongly associated with other immunotherapy-related biomarkers, and it was also an independent risk factor for OS (Table 2). To confirm the advantages of the signature in predicting the prognosis of lung cancer, we compared this signature with other markers by ROC analysis. The prediction performance of this signature was better than TMB, TIDE score, IFNG, merck18, CD8, T-cells dysfunction and exclusion, PD-1, PD-L1, CTLA-4, LAG3, and TIM-3 (Figures 8A,B). Given the limitations of the PCD score’s clinical value in predicting OS in patients with LUAD, a nomogram integrating the PCD score and clinicopathological characteristics was developed to predict 1-, 3-, 5-, and 10-years OS rates (Figure 8C). The subsequent calibration plots suggested that the proposed nomogram had an outstanding performance compared to an ideal model (Figure 8D).

## Discussion

PCD is one of the important mechanisms to regulate cell homeostasis, and its dysfunction often leads to the occurrence of many diseases, including cancer. Programmed cell death includes autophagy, apoptosis, pyroptosis, necroptosis, and ferroptosis. In recent years, molecular prediction models for LUAD have emerged one after another, but most of the models have limited effects (Bai and Duan, 2020; Li and Li, 2020; Li and Long, 2020; Zhao and He, 2020). Moreover, many cell death models only focus on one process of cell deaths, ignoring the crosstalk among cell deaths. In our study, we integrated five classical PCD genes and constructed a prognostic model based on the five PCD genes. Through unsupervised clustering analysis, it was found that differentially expressed PCD genes can well divide LUAD into two clusters. However, there was no difference in OS between the two groups, which may be caused by some genes unrelated to prognosis. Therefore, after excluding the genes unrelated to prognosis, we constructed a risk model of 23 genes most related to prognosis through lasso and multivariate Cox regression analysis. The high- and low-risk groups based on risk score not only have significant differences in OS in all cases but also have strong stratification ability in different LUAD staging groups and clinical subtypes, which was also well verified in three independent GEO validation datasets.

For all 23 genes, they could be roughly classified into apoptosis (UBE4B, HGF, PRKCD, CX3CL1, KRT18, KRT8, PDX1, YWHAG, NOX1, TLR3, FADD, MSX1, BMP5, and CX3CR1), autophagy (PSAP, EEF1A1, HGF, PLK3, PIM2), necroptosis (TLR3, FADD), and ferroptosis (NFS1, GPX2, NOX1, ACSL3, EMC2, PEBP1). Interestingly, we found that four genes (NOX1, FADD, HGF, and TLR3) in the model were involved in multiple cell death pathways.

Nox1 is a homolog of the catalytic subunit of the NADPH oxidase that produces superoxide. In a lung cancer research (Puca and Nardinocchi, 2010), NOX1 can inhibit p53 acetylation, thereby weakening the pro-apoptotic transcription activity of p53. The P53 pathway can induce tumor cell senescence and necrosis through its pro-apoptotic properties, and exert its anti-tumor activity. Ferroptosis is defined as an oxidative, iron-dependent RCD characterized by the accumulation of reactive oxygen species (ROS) and lipid peroxidation products to lethal levels. RAS is the cause of cancer growth, invasion, and metastasis, so some highly aggressive malignancies have been determined to be inherently vulnerable to ferroptosis. Recently, ferroptosis has also been shown to be related to cancer immunotherapy, in which T cells and interferon-γ (IFN-γ) sensitize tumor cells to ferroptosis (Liang and Zhang, 2019). NOX1 is also included in the ferroptosis gene set. The potential of NOX1 as a ferroptosis-related predictor has been analyzed in many cancers (Zhu et al., 2021; Zhu and Yang, 2021). In the rhabdomyosarcoma cell (Dächert and Ehrenfeld, 2020) and non-small lung cancer cell model, NOX1 can affect lipid peroxidation, ROS production, and other aspects to participate in ferroptosis signal transduction.

TLR3 has been regarded as a tumor suppressor-related factor in many lung cancer studies. In *in vitro* experiments, it has been found that TLR3 activation induces apoptosis of lung cancer cell lines (Bianchi and Alexiadis, 2020). The emergence of this tumor suppressor function is that TLR3 can promote the apoptosis of cancer cells on the one hand, and on the other hand, it is also related to the induction of cytotoxic factors and the production of some interferons (Lau and Zhu, 2017). However, in our data, although the results were not significant, TLR3 did show a cancer-promoting effect. In fact, with the deepening of research, the effect of TLR3 tends to be a “two-side effect”, that is, TLR3 can promote and inhibit cancer at the same time. However, TLR3 may be associated with tumor progression, metastasis, and treatment resistance, resulting in poor prognostic outcomes (Muresan and Bouchal, 2020). In the lung environment, the TLR3 of lung epithelial cells can be activated by exosomal RNA derived from the primary tumor to stimulate the NF-κB, ERK, and p38 pathways to secrete chemokines. The production of these chemokines has significance for the construction of the environment before tumor metastasis (Liu and Gu, 2016).

Among them, the HGF gene is not only involved in autophagy but also one of the apoptosis-related genes. Mesenchymal epithelial transforming factor (MET) and liver growth factor receptor (HGF) pathway signals mediate wound healing and liver regeneration under normal physiological conditions. When the HGF/MET pathway is dysregulated, it participates in mediating proliferation, apoptosis, and migration, and induces a variety of cancers (Salgia, 2017; Liang and Wang, 2020). Compared with MET, the potential of HGF as a prognostic indicator of lung cancer is significantly smaller and controversial (Moosavi and Giovannetti, 2019). In our research, HGF acts more as a protective factor to inhibit the progression of lung cancer. As for how HGF affects the occurrence of lung cancer by regulating the cell death program, its internal mechanism is still insufficiently studied.

FADD (Fas Associated Via Death Domain) can be recruited by TNFRSF6/Fas-receptor, tumor necrosis factor receptor, TNFRSF25, and TNFSF10/TRAIL-receptor, and thus it participates in the death signaling initiated by these receptors. FADD can be combined with TAT to rapidly constitute DISC (death-inducing signaling complex) assembly. TAT-FADD inhibits the initiation of classic NLRP3 inflammasomes and limits the processing and secretion of pro-inflammatory IL-1β, thereby regulating the anti-apoptotic and pro-inflammatory NF-κB signal activation in cancer cells (Ranjan and Waghela, 2020). In lung adenocarcinoma, FADD, a ferroptosis-related gene, was also identified as a predictor (Wang and Wu, 2021).

To explore the potential mechanism to the significant difference of OS in different risk groups, we further explored the biological processes in high-risk and low-risk groups through GO, KEGG, and GSEA analysis. Our results showed that the high-risk group was significantly enriched in the processes of metabolism and cell adhesion, including Amino Sugar and Nucleoside Sugar Metabolism, Ascorbate and Aldarate Metabolism, Galactose Metabolism and Cells Adhesion Molecules (CAMs).

Glucose, glutamine, fatty acids, and amino acids are the main drivers of tumor growth. Therefore, metabolic changes and cell energy imbalance are now considered to be the markers of all cancers (Akella and Ciraku, 2019). There is a complex relationship between the immune system and cancer, and the immune system plays a dual role in tumor development. Immune system effector cells can recognize and kill malignant cells, and immune system-mediated inflammation can also promote tumor growth and regulate cell inhibition of anti-tumor response. The core of all anti-tumor responses is the ability of immune cells to migrate to the tumor site and interact with malignant cells, where CAMs are essential in mediating these processes (Harjunpää et al., 2019). This research proposed that cell-to-cell interaction and cell adhesion are key mediators of cancer progression, including immune evasion and metastatic transmission (Läubli and Borsig, 2019). Therefore, the reason for the lower OS in the high-risk group may be regulated by these pathways. However, the low-risk group was enriched in multiple immune pathways, including Natural Killer cell-mediated cytotoxicity, B cell, T cell receptor signaling pathway, Lymphocyte mediated immunity. T cells, B cells, dendritic DC cells, and natural killer cells are anti-tumor immune cells and play a crucial role in anti-tumor (Corrales and Matson, 2017; Chiossone and Dumas, 2018). In contrast, T (and B or DC) cell-mediated tumor immunity was significantly enriched in the low-risk group. Moreover, the low-risk group had higher immune cell infiltration, immune pathways, immune score, and lower tumor purity. These results showed that the PCD-based signature presented unique characteristics in terms of a biological pathway, immune cell infiltration, and immune inflammation profile in the tumor microenvironment (TME).

With the in-depth study of tumor immunology and molecular biology, ICBs provide a new way for the treatment of tumors, especially in advanced lung cancer. The study of ICIs targeting CTLA-4, PD-1, and PD-L1 is booming, and clinical trials have proven their efficacy and safety (Chae and Arya, 2018; Huang and Jiang, 2021). In this study, the expression levels of PD-1 and CTLA4 were significantly up-regulated in the low-risk group with a better prognosis, suggesting that these patients may be sensitive to ICIs targeting PD-1 and CTLA4. To test this conjecture, we compared the relationship between the PCD based risk score and the markers of immunotherapy response and found that the risk score was positively correlated with TIDE score and T cells exclusion score, but negatively correlated with T cells dysfunction score, Merck 18, IFNG and CD8. TMB, PD-L1, and TIDE were newly discovered predictors of immunotherapy response. In particular, TIDE has been shown to have better prognostic performance than other biomarkers or indicators. It was confirmed that the lower TIDE score showed higher response rates to both anti-PD-1 and anti-CTLA-4 inhibitors, and merck18 (T-cell inflamed signature) can contribute to T-cell dysfunction (Woo and Corrales, 2015). Therefore, this PCD-based signature identified low-risk LUAD patients should be suitable for ICBs treatment because of their lower TIDE score and T cell dysfunction score. To verify the superiority of the signature, we compare the prognostic ability of the signature-based on PCD with other indicators. The ROC curve showed that our signature had better prediction performance than other biomarkers. In addition, we constructed a nomogram to predict the OS of LUAD patients, which showed a power predictive value in predicting the OS of LUAD.

Although the PCD-based signature may be utilized as an independent prognostic factor and can predict immunotherapy response in LUAD, there were several limitations to this study. First and foremost, because all four cohorts are retrospective data sets, a prospective analysis of this PCD-based signature is required. Second, all expression data was obtained from a public dataset, and the results must be confirmed using new methodologies and fresh specimens. Third, we evaluated the potential to predict immunotherapy response indirectly, and more study is needed to confirm this conclusion.

## Conclusion

In summary, our comprehensive analysis of PCD-based signature showed a diverse set of regulatory mechanisms by which they influence the tumor immunological landscape, clinicopathological characteristics, and prognosis. We also discovered the importance of PCD-based signature in immunotherapy. These results emphasize the clinical importance of PCD-based signature and propose innovative thinking for guiding individualized immunotherapy for LUAD patients.

## Data Availability

The original contributions presented in the study are included in the article/Supplementary Material, further inquiries can be directed to the corresponding author.

## Abbreviations

PCD, Programmed cell death; LUAD, lung adenocarcinoma; TME, tumor microenvironment; TCRs, T cell receptors; TCGA, The Cancer Genome Atlas; GEO, Gene Expression Omnibus; GSEA, Gene Set Enrichment Analysis; KEGG, Kyoto Encyclopedia of Genes and Genome; GO, Gene Ontology; ssGSEA, single sample GSEA; GSVA, Gene Sets Variation Analysis; TMB, tumor mutational burden; TCIA, The Cancer Immunome Atlas; TIDE, Tumor immune dysfunction and exclusion; CNV, copy number variation; PCA, Principal component analysis; t-SNE, t-distributed stochastic neighbor embedding analysis ; ROS, reactive oxygen species; IFN-γ, T cells and interferon-γ; MET, Mesenchymal epithelial transforming factor; HGF, CAMs, Galactose Metabolism and Cells Adhesion Molecules; ICIs, checkpoint inhibitors.
