# Supplementary material for: Comprehensive Analysis of Programmed Cell Death Signature in the Prognosis, Tumor Microenvironment and Drug Sensitivity in Lung Adenocarcinoma
Source: Front Genet. 2022 May 18;13:900159. doi: 10.3389/fgene.2022.900159 (PMC9157820; doi:10.3389/fgene.2022.900159)
Supplement: Supplementary file 1 [file Table1.DOCX]

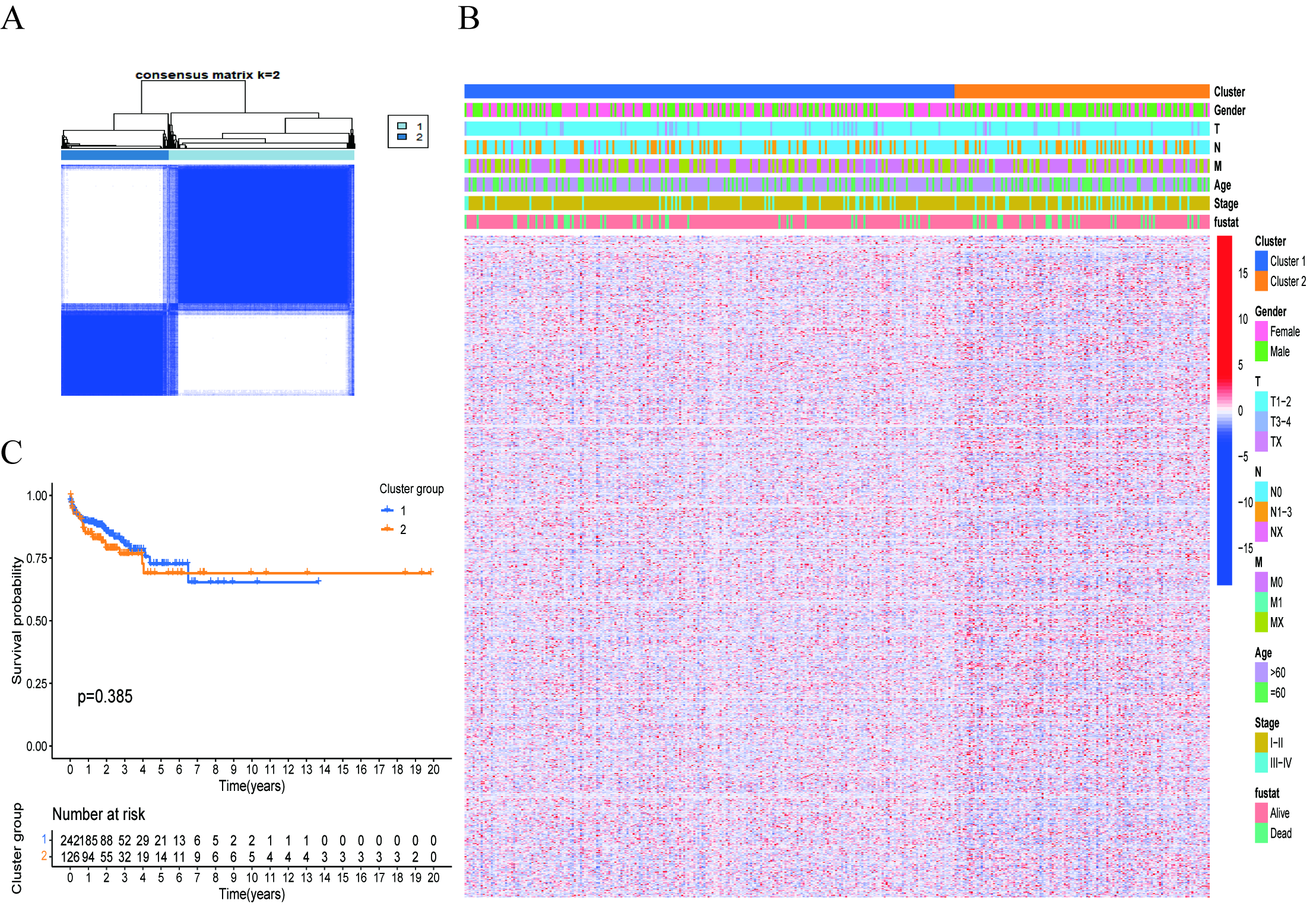


**Figure1**. Unsupervised clustering analysis in TCGA dataset. (A) The TCGA dataset were stratified into two clusters based on the consensus matrix(k=2). (B) Differences

in clinicopathologic features and expression levels of differentially expressed PCDs between the two distinct clusters. (blue: low expression level; red: high expression level). (C) Survival curve analysis show no difference in OC between the two clusters (P>0.05) .

`
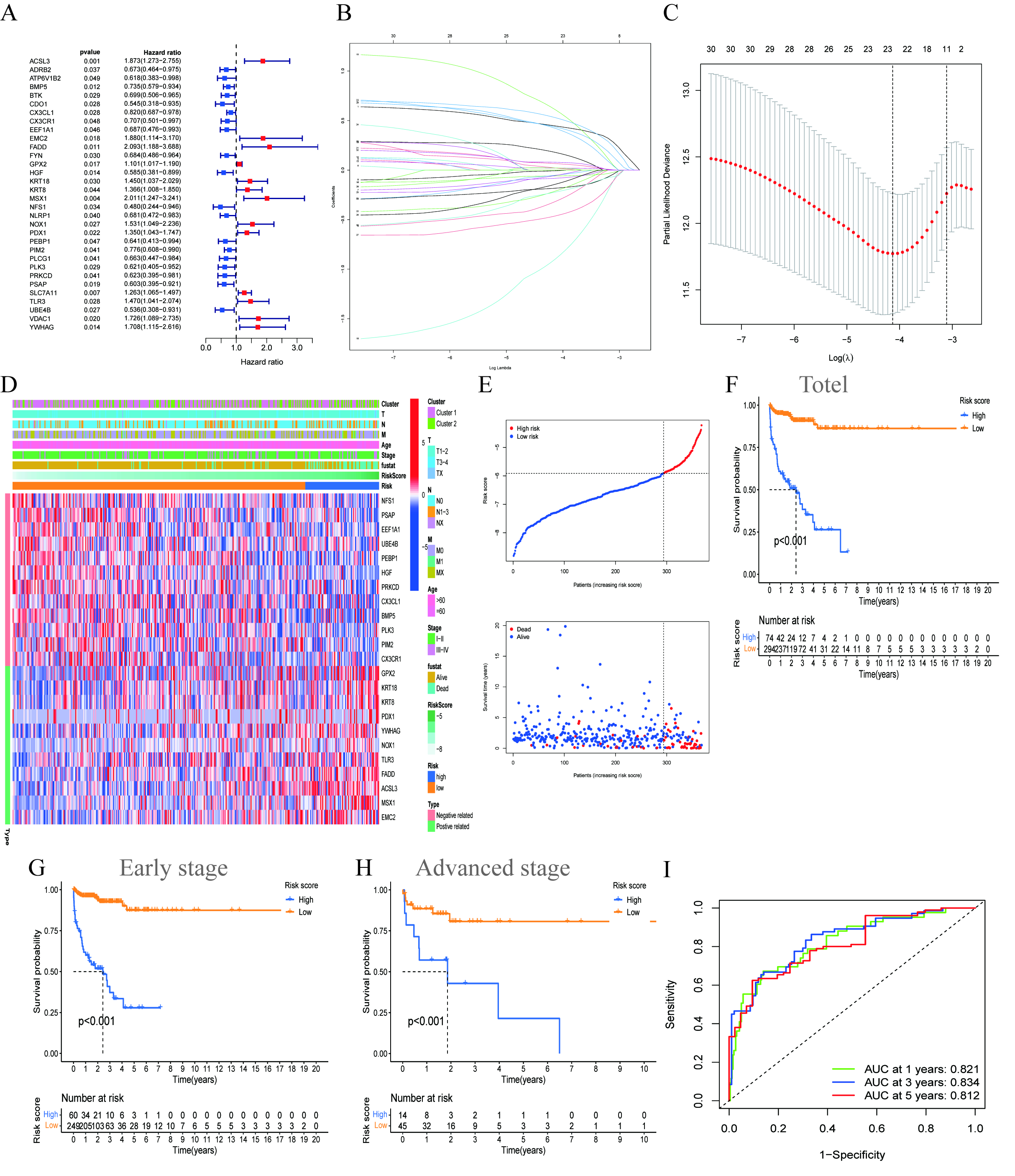


**Figure2**. Construction of the PCD-based signature in TCGA datasets. (A)The forest map showing the 32 prognostic PCD-related DEGs by univariate cox regression analysis (P<0.05). (B) Cross-validation for adjusting the parameter selection in the LASSO regression. (C) LASSO regression analysis of the 32 OS-related DEGs. (D)Heatmap showing the features of 23 identified genes and clinical characteristics of two molecular clusters. (E) The distribution of risk score and survival states. Kaplan Meier plot showing the significant difference of OS in total LUAD (n=368)(F) ;in early-stage (I or II) (n=309) (G)and in advanced stage (III or IV) (n=59) (H) in the high-risk and low-risk groups. (I) ROC curve showing the sensitivity and specificity to predict 1-, 3-, and 5-year survival based on PCD-based signature, with the area under curve being 0.821,0.834, and 0.812, respectively.


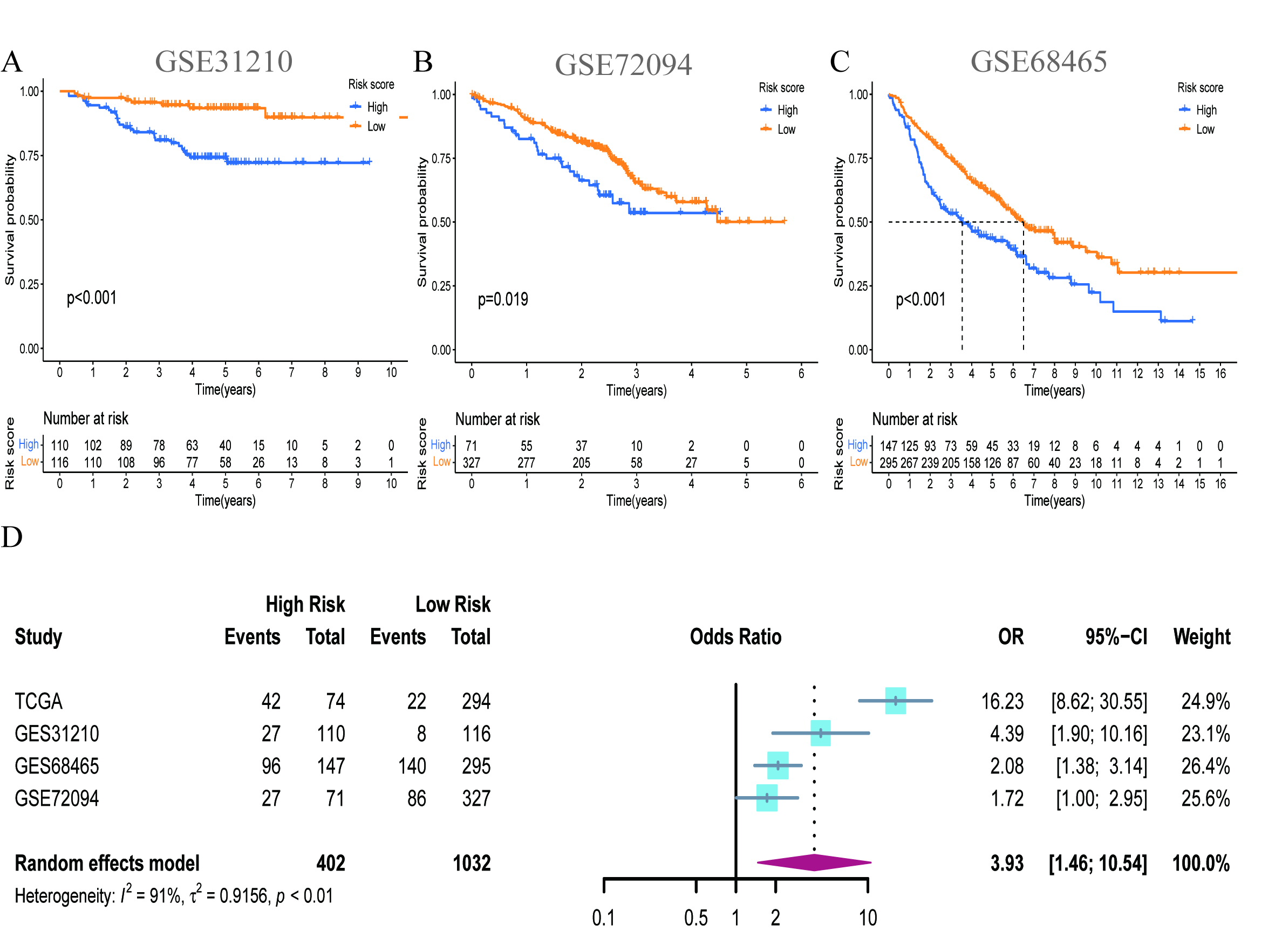


**Figure3**. Validation of the PCD-related signature in GEO datasets. (A)Kaplan-Meier curves showing the significance of OS in GSE31210(n=226);(B) in GSE 72094 (n=398) and (C) in GSE68465(n=443). (D)A meta-analysis showed the prognostic outcomes in four independent datasets(HR:3.93; 95%CI:1.46-10.54).


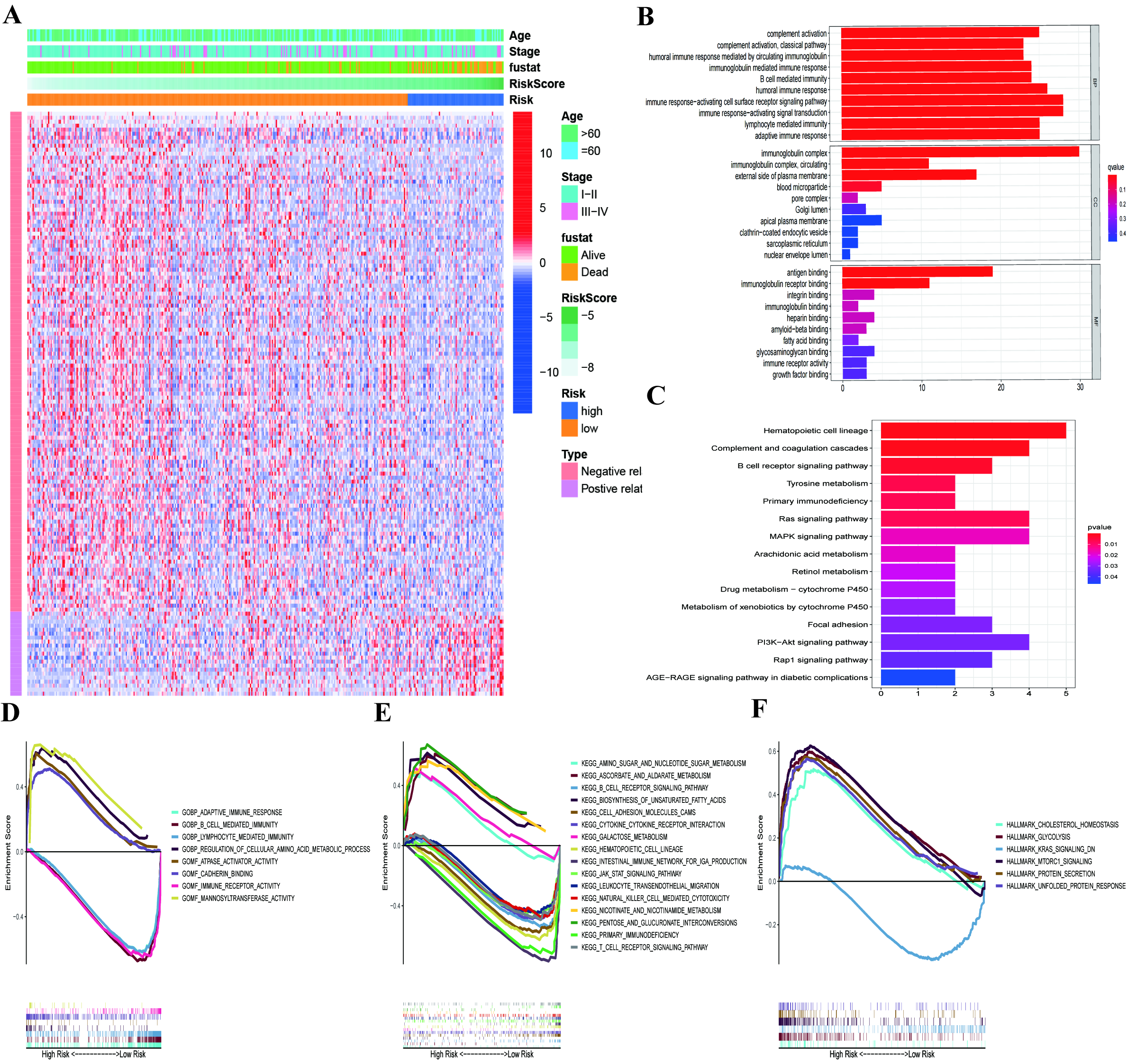


**Figure4**. Biological pathway and function between the two risk groups. (A) Heatmap and the difference of clinical characteristics and DEGs between the high- and low-risk groups(blue: low expression level; red: high expression level). (B)GO and (C)KEGG analysis revealed that DEGs in high- and low-risk subgroups were enriched in multiple immune and tumor-related pathways. (D-F) GSEA analysis validated multiple biological pathways related to inmmunity and tumor enriched in the high-risk group and low-risk group.


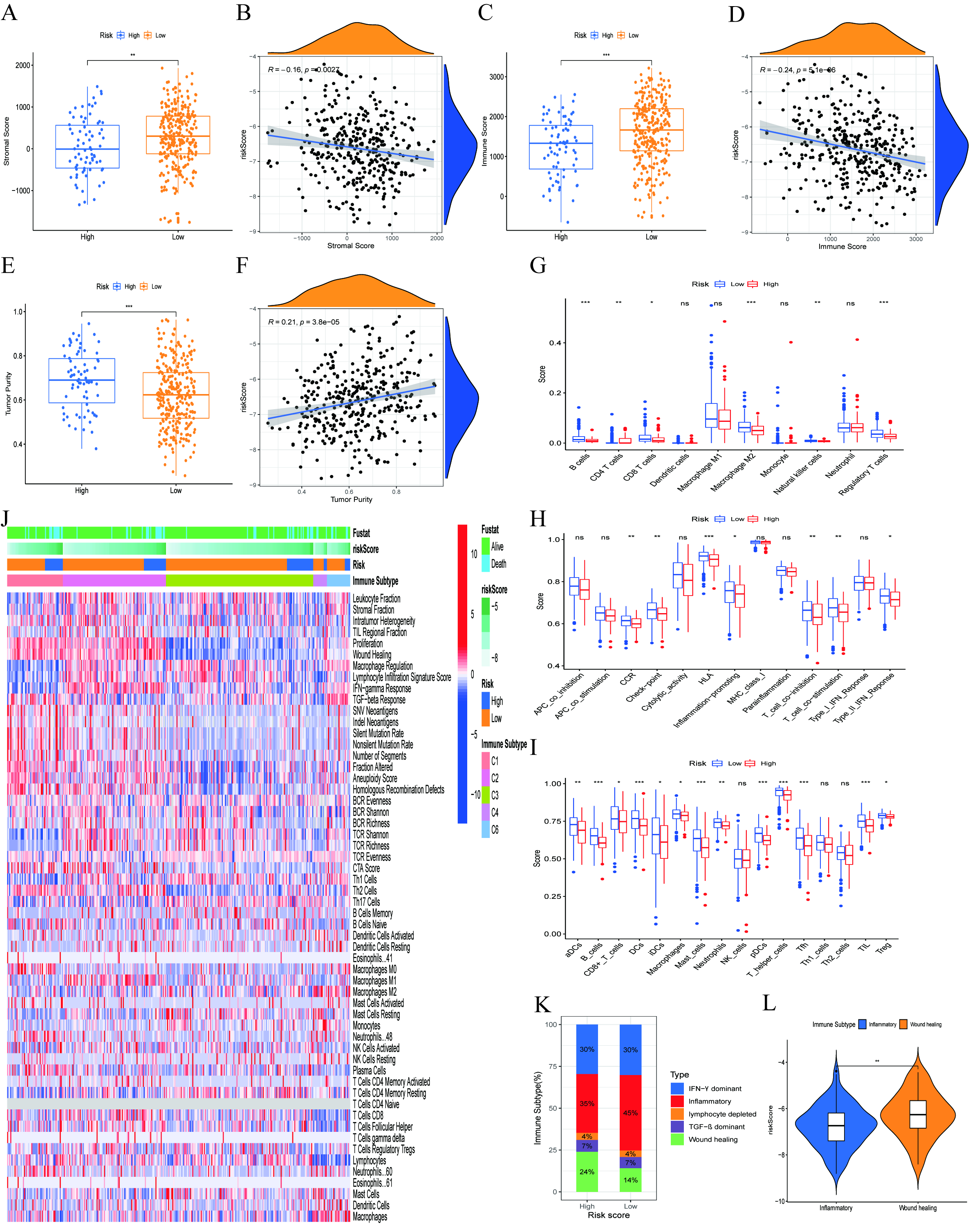


**Figure5**. The immune landscape of PCD-related signature in LUAD. (A-B) Significant differences of stromal score in high- and low- risk groups and correlation with risk score. (C-D) Significant differences of immune score in high- and low- risk groups and correlation with risk score. (E-F) Significant differences of tumor purity in high- and low- risk groups and correlation with risk score. (G) Comparison of 11 types of immune cells in high- and low-risk groups through CIBERSORT. (H-I) Comparison of 16 types of immune cells and 13 immune-related pathways in high-risk and low-risk groups through ssGSEA. (J)The heatmap showing different cell type panels in different immune subtypes of lung cancer. (K) Estimated immune subtype proportion in high-risk and low-risk groups. (L) Comparison of the risk score in inflammatory subtype and wound healing subtype. (*, **, ***, and **** represent P < 0.05, P < 0.01, P < 0.001 and P < 0.0001, respectively)


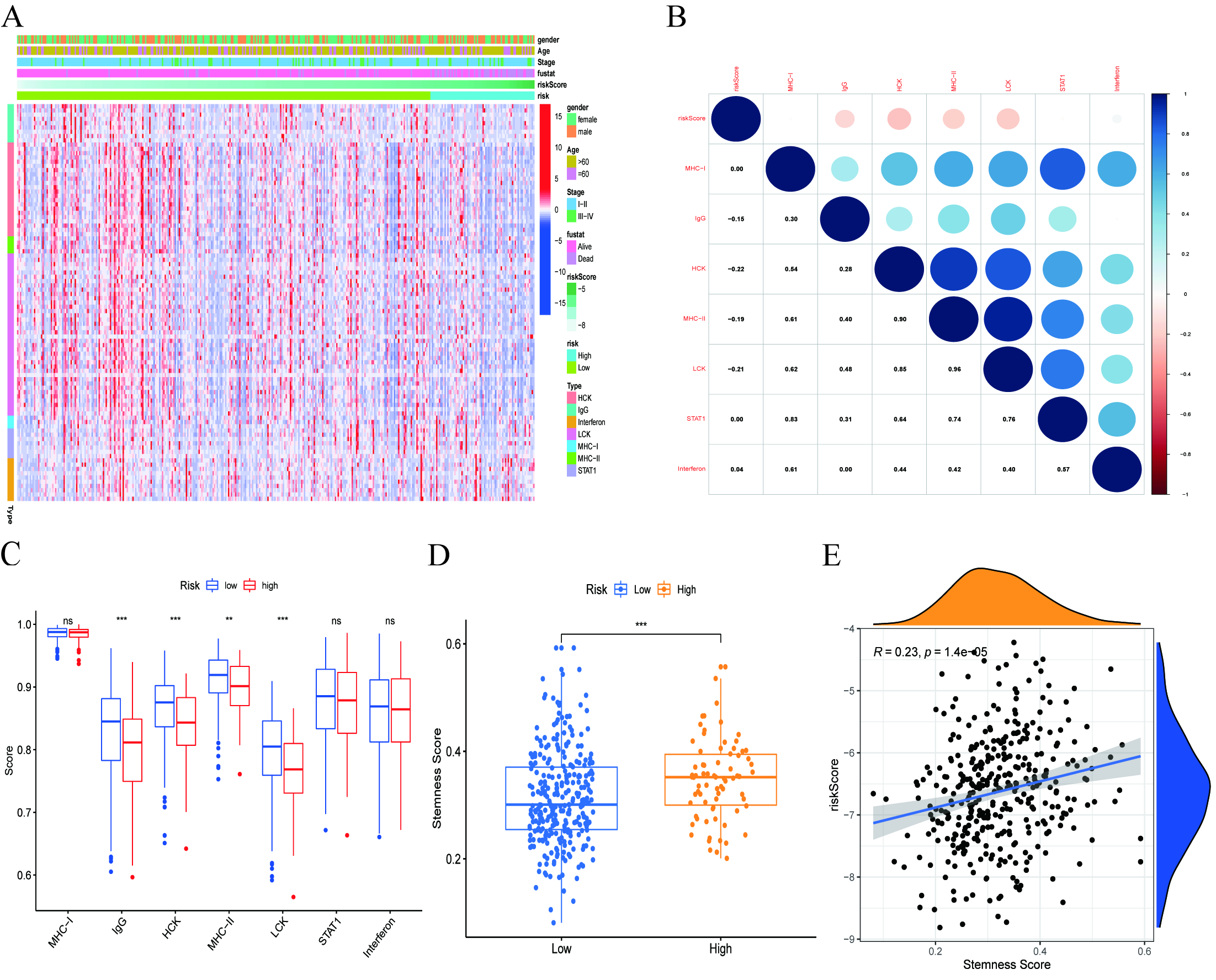


**Figure6**. Inflammatory and immunologic profile of the PCD-related signature in TCGA cohort. (A) Heatmap showed the gene expression of immune-inflammatory metagenes in the two risk groups(blue: low expression level; red: high expression level). (B) A correlogram was generated based on Pearson P-value between risk score and metagenes. (C) Comparison of the immune-inflammatory metagenes in high-risk and low-risk groups. (D) Comparison of the stemness score in high-risk and low-risk groups. (E) Correlation of stemness score and risk score(R=0.23, P<0.05). (*, **, ***, and **** represent P < 0.05, P < 0.01, P < 0.001 and P < 0.0001, respectively)


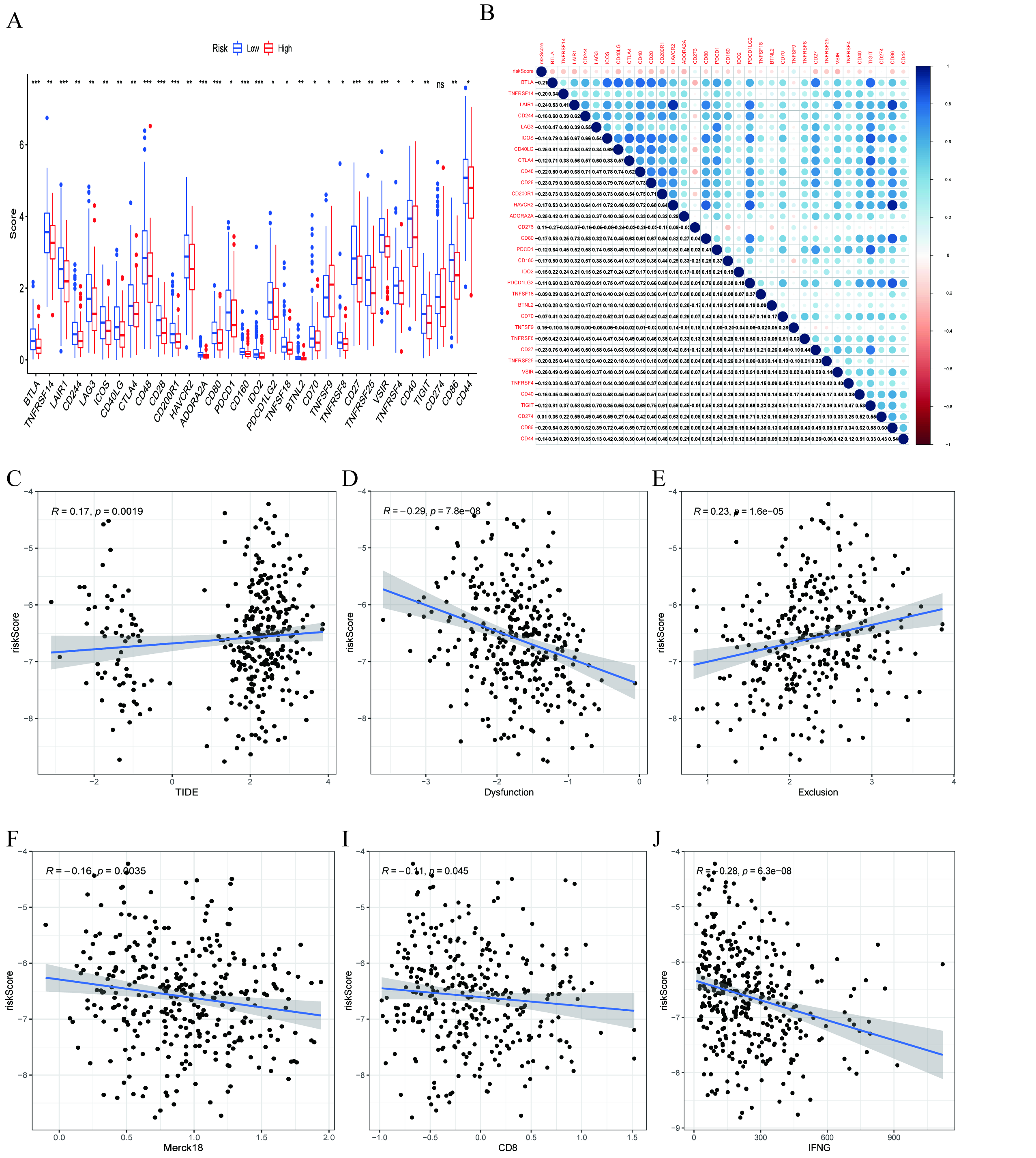


**Figure7**. Relationship between the PCD-related signature and biomarkers of immunotherapy. (A) Comparison of the immune checkpoints in high-risk and low-risk groups. (B) A correlogram was generated based on the Pearson P-value between risk score and immune checkpoints. (C) Correlation of risk score and TIDE score; (D) T cells dysfunction score; (E)T cells exclusion score; (F) Merck18 score; (I) CD8; (J) IFNG in high-risk and low-risk groups. (*, **, ***, and **** represent P < 0.05, P < 0.01, P < 0.001 and P < 0.0001, respectively)


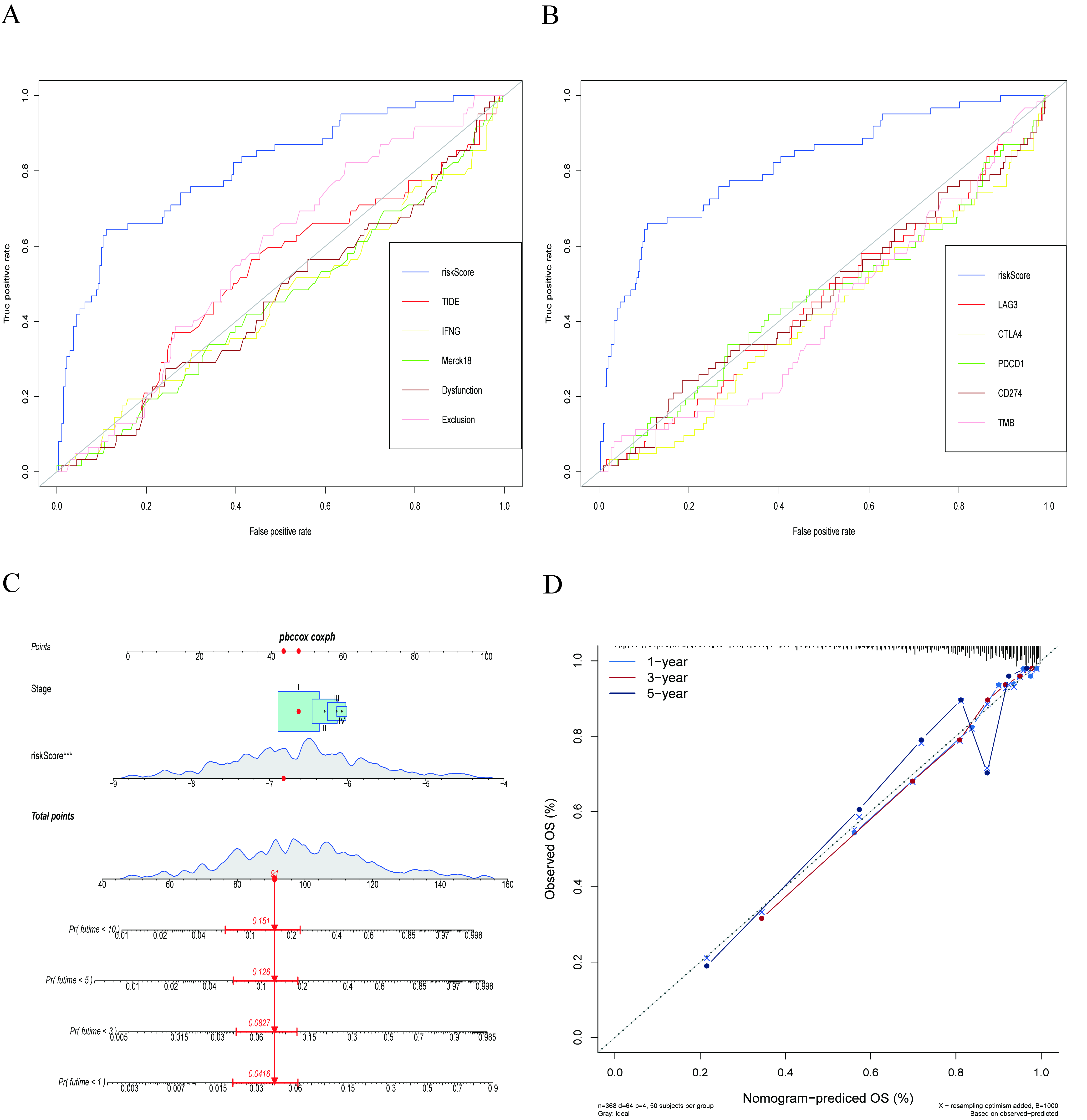


**Figure8**. Comparison of the predictive power of PCD-related signature with other biomarkers and construction of a nomogram in TCGA cohort. (A-B) ROC curve compared the sensitivity and specificity of risk score and other biomarkers for predicting OS. (C) Nomogram combined the TNM staging and risk score for predicting the 1-, 3-, 5-, and 10-year OS . (D) Calibration curves of the nomogram for predicting of 1-, 3-, and 5-year OS.
