# Supplementary material for: Comprehensive Analysis of Programmed Cell Death Signature in the Prognosis, Tumor Microenvironment and Drug Sensitivity in Lung Adenocarcinoma
Source: Front Genet. 2022 May 18;13:900159. doi: 10.3389/fgene.2022.900159 (PMC9157820; doi:10.3389/fgene.2022.900159)
Supplement: Supplementary file 2 [file Table2.DOCX]

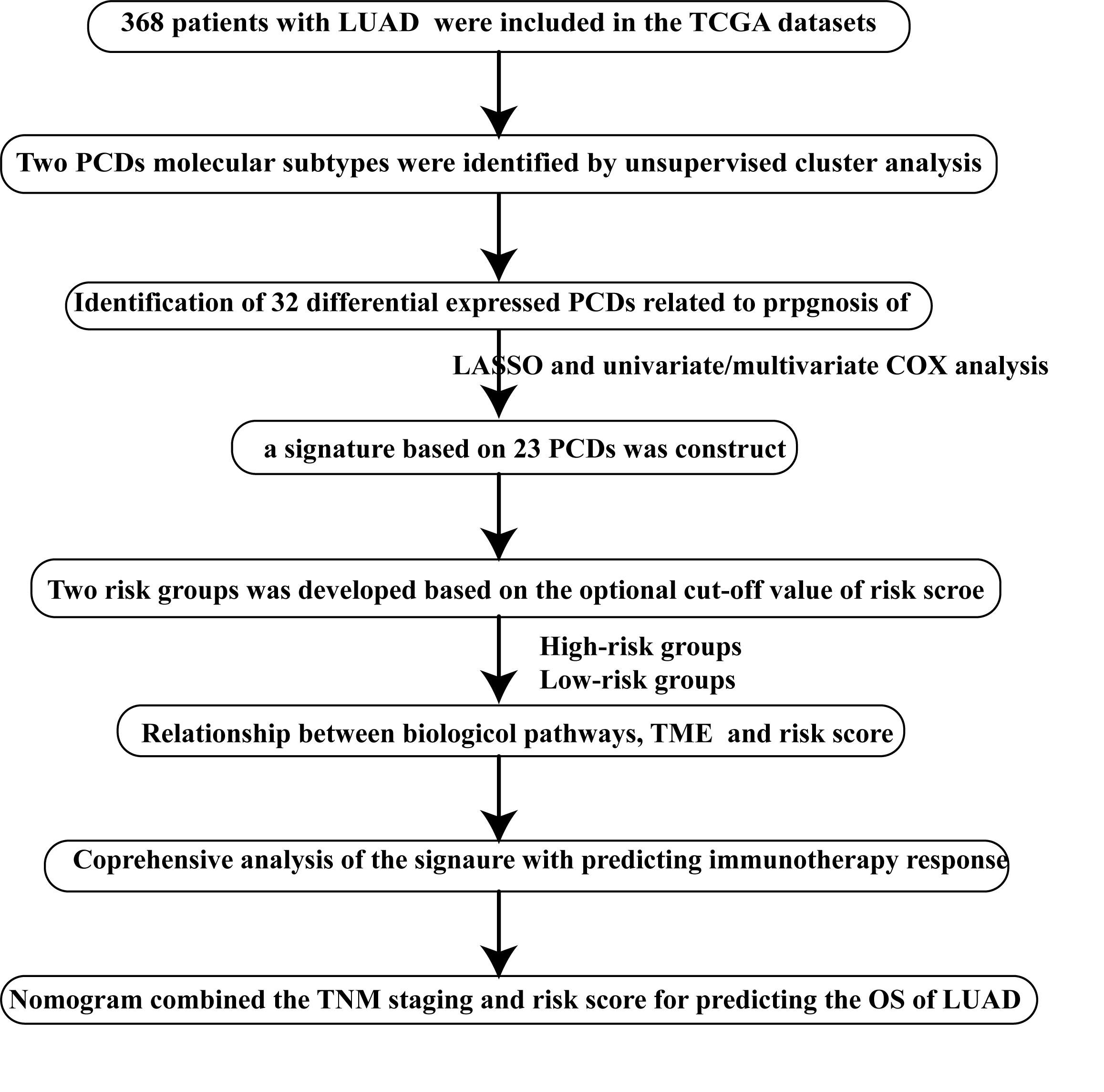


**Supplementary Fig.S1**.The flow diagram showed the entire analytical process of the study


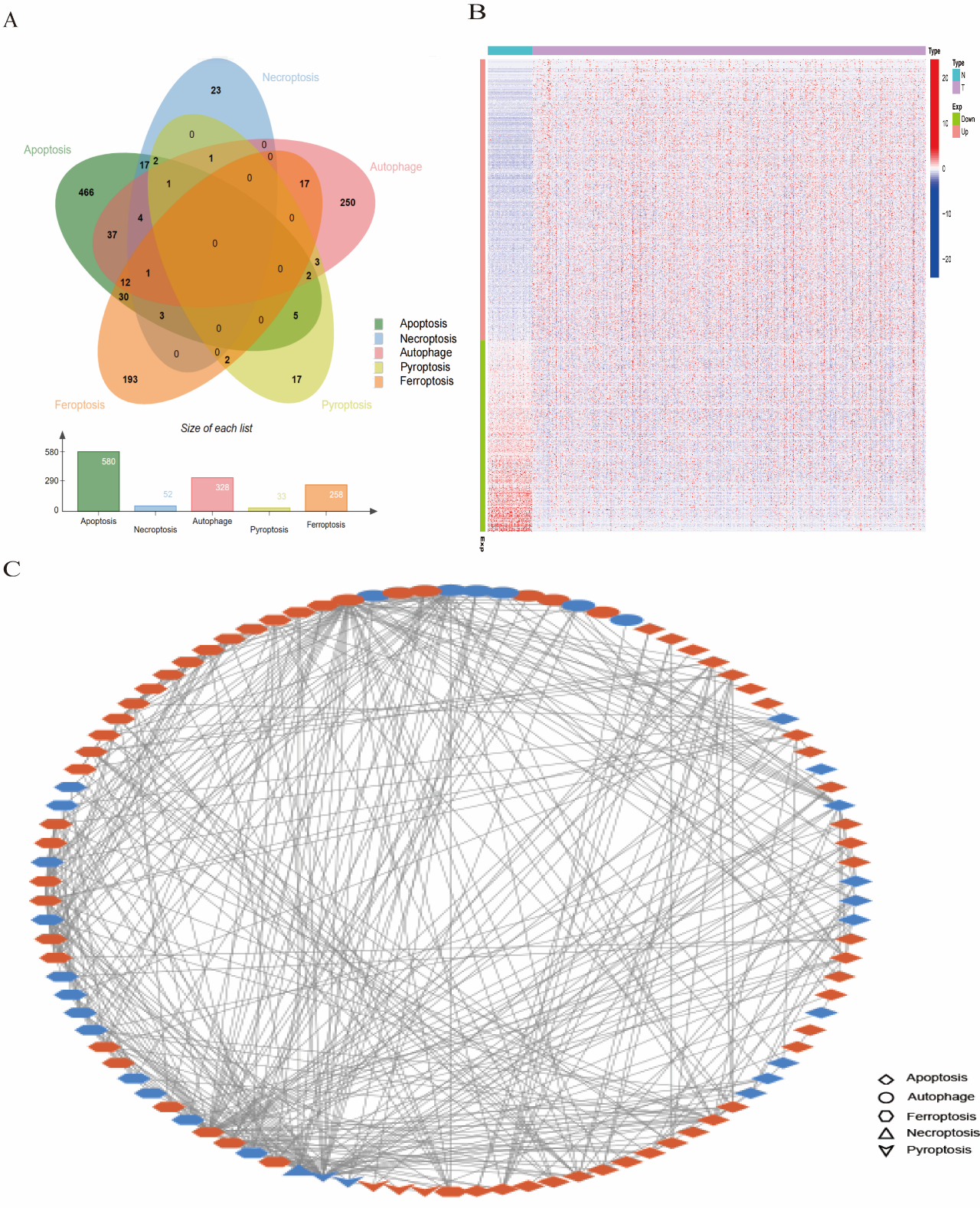


**Supplementary Fig.S2**. Identification of PCDs in LUAD.(A) Venn diagrams of five PCD gene sets, namely apoptosis, necroptosis, autophagy, pyroptosis, and ferroptosis. (B) Heatmap showed DEGs between tumor and normal tissue(N:normal, T: tumor; blue: low expression level; red: high expression level) . (C) Gene regulatory network of selected genes(fold change≥2). Each dot represents one gene and different shapes represent different sources. Red represents up-regulate gene and blue represents down-regulated gene.


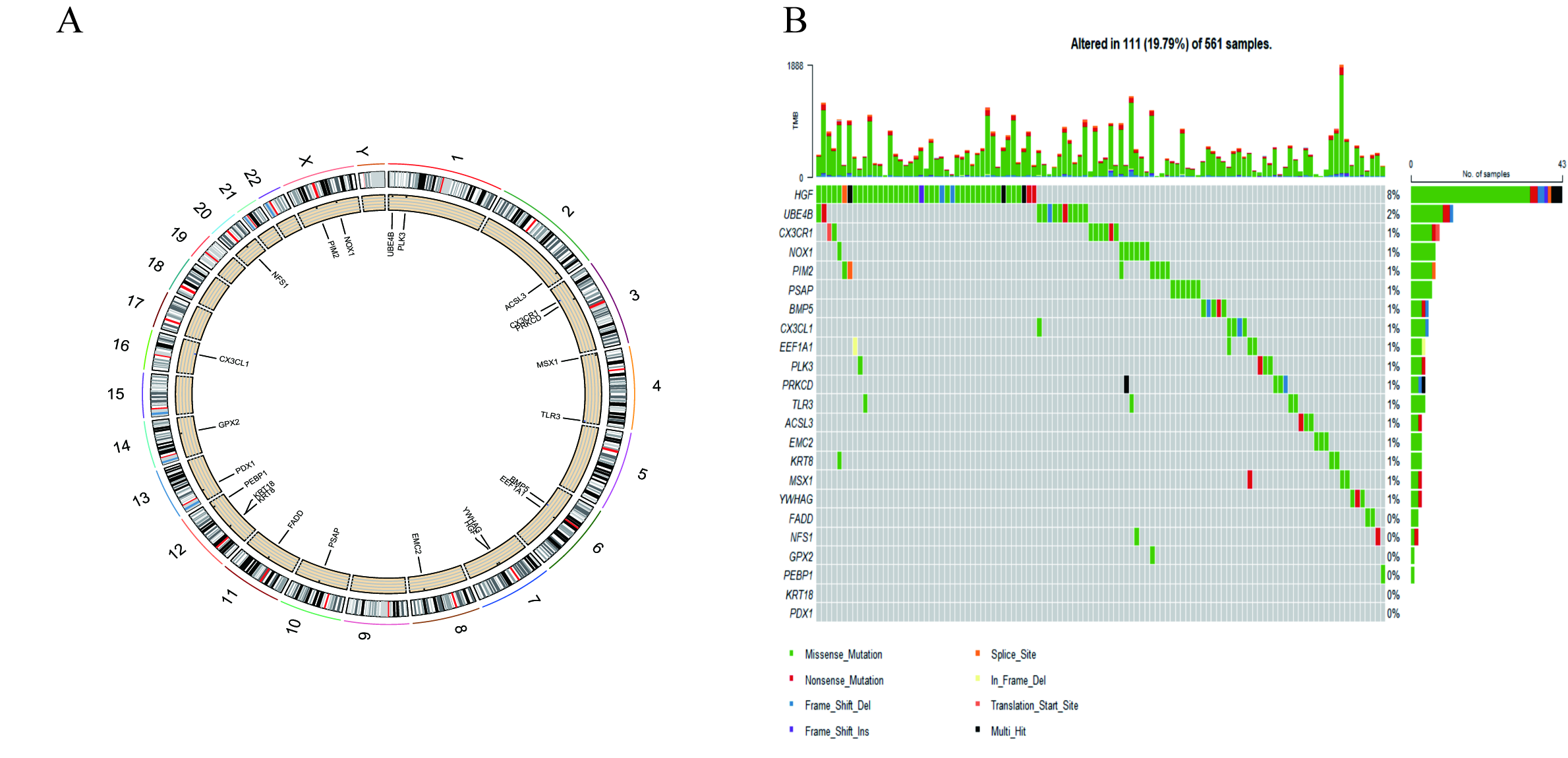
**Supplementary Fig.S3**. The chromosomal location and mutation information of 23 genes in PCDs-based signature. (A). Locations of CNV alterations in these 23 genes on 23 chromosomes. (B) Mutation frequencies of these genes in 561 patients with LUAD in TCGA cohort


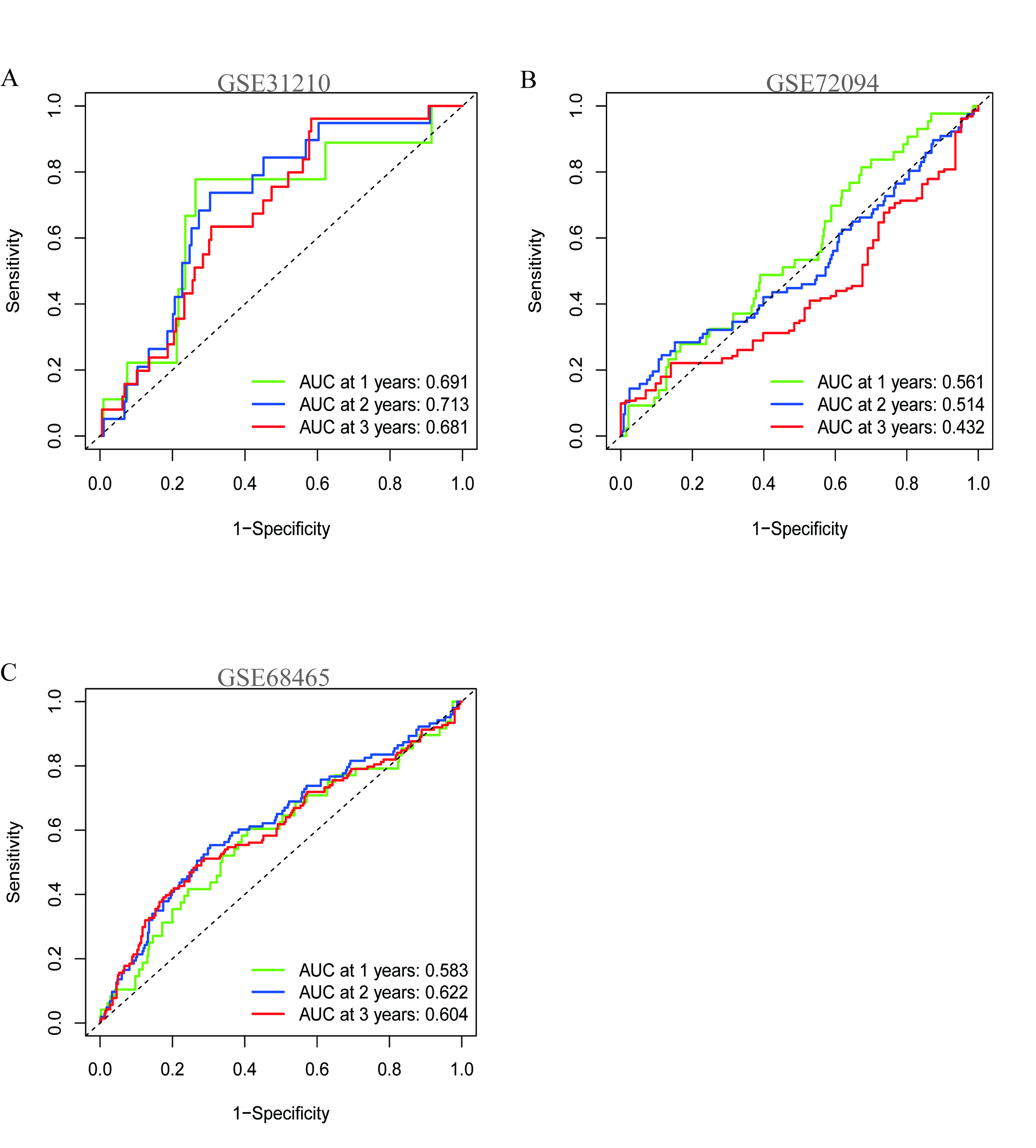


**Supplementary Fig.S4**. ROC curves showed the predictive efficiency of the risk score: ROC curves for 1,2,3 years in GSE31210 datasets (A);in GSE72094 datasets (B); and in GSE68465 dataset (C).

**Supplementary Fig.S5**. Verification of the prognostic value of PCD-related signature in clinical subtypes in the TCGA cohort. OS for patients with (A)Age>60 years. (B)Age≤60 years.(C)Female. (D)Male. (E)T1-2 stage. (F)T3-4 stage. (G)N0 stage. (H)N1-3 stage. (I)M0 stage. (I)M1 stage.


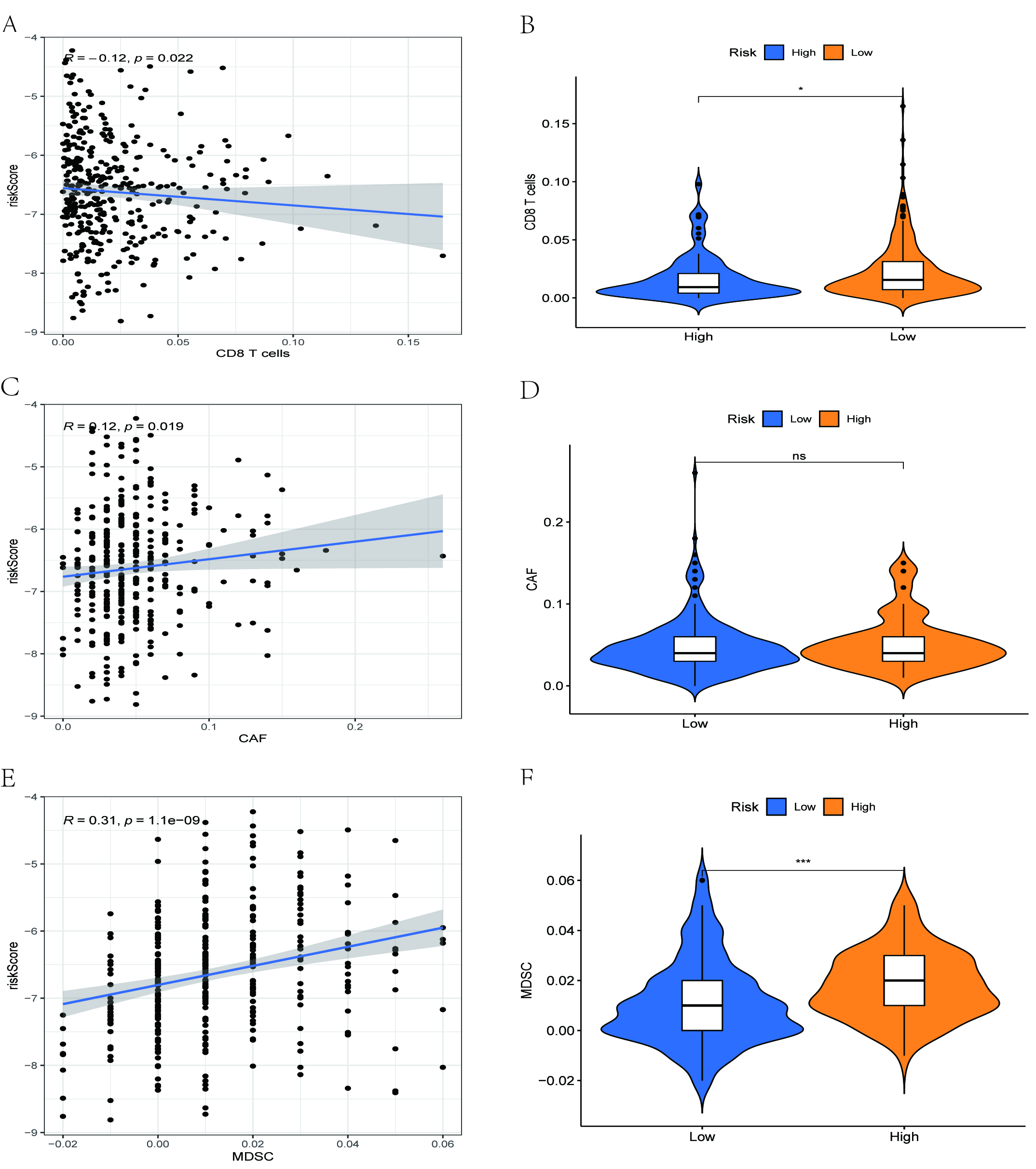


**Supplementary Fig.S6** Relationship between the PCD-related signature and immune cells. (A) The correlation between CD8^+^ T cells and risk score. (B)The distribution of CD8^+^ T cells in the high- and low-risk groups. (C) The correlation between CAF and risk score. (D)The distribution of CAF in the high- and low-risk groups. (E) The correlation between MDSC and risk score. (F)The distribution of MDSC in the high- and low-risk groups. (*, **, ***, and **** represent P < 0.05, P < 0.01, P < 0.001 and P < 0.0001, respectively).


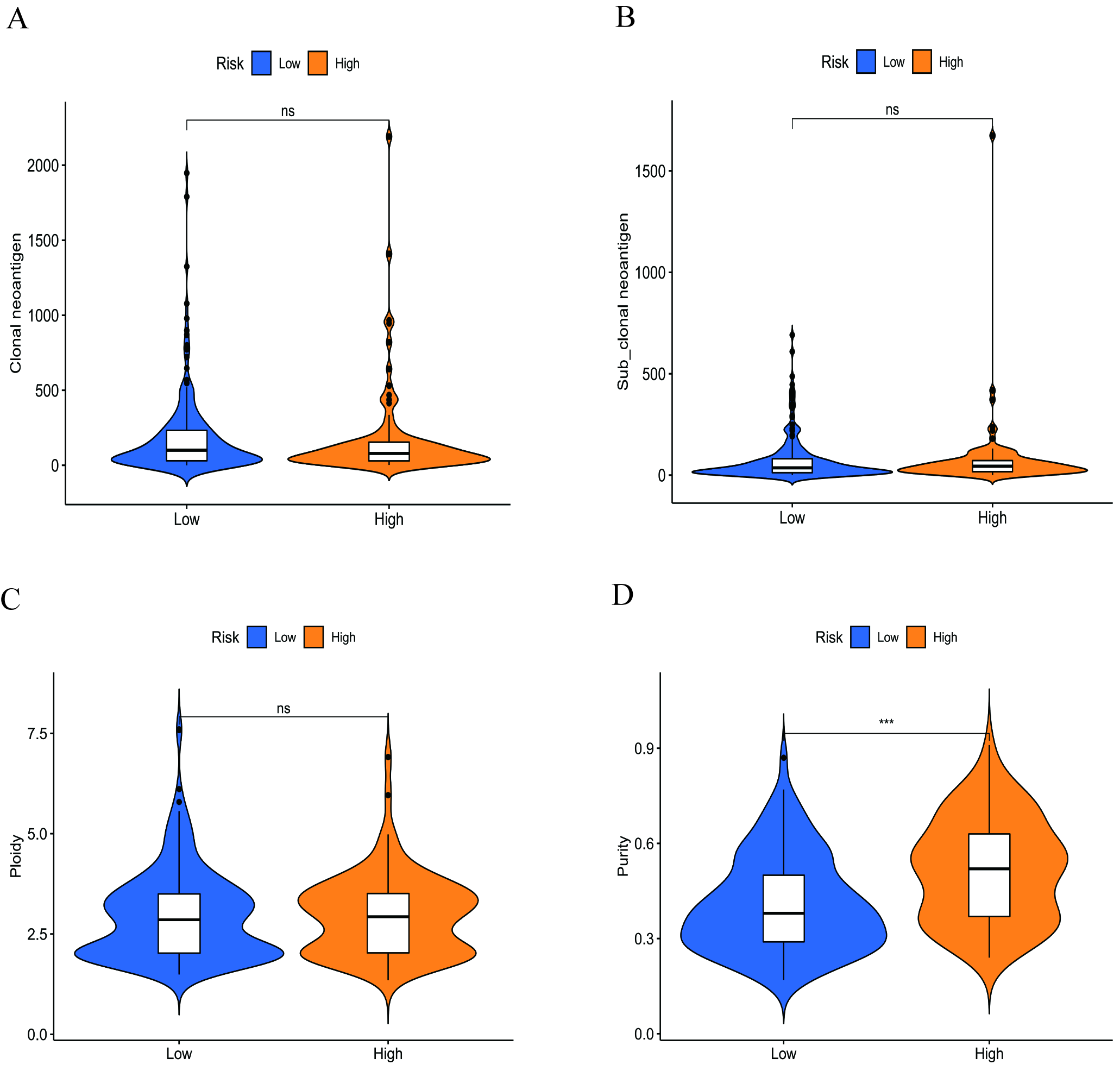


**Supplementary Fig.S7**. Distribution of tumor neoantigens and purit***y*** in high-risk and low-risk groups. The distribution and comparison of (A)Clonal. (B)Sub-clonal. (C) Ploidy. (D)Purity. (*, **, ***, and **** represent P < 0.05, P < 0.01, P < 0.001 and P < 0.0001, respectively).

**Supplementary Fig.S8.** Difference of somatic mutation in the high-risk and low-risk group. (A,B) A waterfall map showed the somatic mutation features in the high- and low- risk groups. Each column represent a particular patient. TMB was depicted in the bar chart above, and the number on the right denotes the mutation frequency of each gene. The ratio of each type of variation was shown in the bar chart on the right. (C)Correlation of risk score and TMB. (D) Comparison of TMB in the high- and low-risk groups.

**Supplementary Fig.S9.** The comparison of estimated IC50 of chemical drugs in high and low-risk groups.
